# Respiratory pattern change in female and male runners by respiratory tract restriction using a respirator

**DOI:** 10.1371/journal.pone.0353784

**Published:** 2026-07-16

**Authors:** Petr Bahenský, Tomáš Mrkvička, David Marko, Renata Malátová, Miroslav Krajcigr, Eric Harbour

**Affiliations:** 1 Department of Sports Studies, Faculty of Education, University of South Bohemia in České Budějovice, České Budějovice, Czech Republic; 2 Department of Data Science and Computing Systems, Faculty of Agriculture and Technology, University of South Bohemia in České Budějovice, České Budějovice, Czech Republic; 3 Department of Sport and Exercise Science, Paris Lodron University of Salzburg, Salzburg, Austria; Federal University of Pernambuco: Universidade Federal de Pernambuco, BRAZIL

## Abstract

Respiratory protective equipment, particularly filtering facepiece respirators (FFRs), are increasingly used during physical activity, yet their influence on breathing mechanics remains unclear. This randomized, crossover trial investigated the impact of N95 FFRs on thoracoabdominal breathing patterns during graded treadmill exercise. Twenty competitive runners (10 male, 10 female; 18.9 ± 2.3 years) performed Bruce protocol tests with and without FFRs, while optoelectronic plethysmography quantified the contributions of pulmonary ribcage (RCp), abdominal ribcage (RCa), and abdomen (Ab) to tidal volume. Global ventilatory parameters (tidal volume, breathing frequency, minute ventilation) were unaffected by FFR use. However, compartmental analysis revealed significant alterations in breathing patterns at higher intensities. At 12 and 15 minutes, FFRs reduced RCp contribution (−7.85 ± 2.49%, P = 0.002; −8.41 ± 3.35%, P = 0.012) and increased Ab contribution (+5.97 ± 2.08%, P = 0.004; + 6.51 ± 2.80%, P = 0.020). Across all phases, females demonstrated moderate, but variable RCp dominance (P = 0.052). These findings indicate that while FFRs do not impair overall ventilation in trained runners, they substantially modify the biomechanics of breathing under load, reflecting compensatory adjustments that likely help preserve ventilatory efficiency. The results suggest that well-trained athletes may tolerate acute respiratory stress more effectively than the general population, which has implications for athletic training, rehabilitation, and occupational health where respirator use is required.

**Trial registration:**

ClinicalTrials.gov NCT07032740

## 1. Introduction

The quality of breathing is important for each individual, and it is crucial for endurance athletes. Effective ventilation is directly involved in the performance achieved, as it affects oxygen supply, metabolic capacity and fatigue resistence [1]. Optimal breathing mechanics is essential to maintain performance in endurance athletes, with the distribution of breathing work between the chest compartments playing an important role in preventing respiratory muscle fatigue [1,2]. For competitive runners, optimal breathing mechanics are essential [3] since they may affect cognitive function, decision making and concentration [4,5], and overall physical performance [6–8]. Even minor changes in ventilation patterns – for example, those induced by external respiratory aids – can significantly affect performance [9].

During the COVID-19 pandemic, the obligatory use of respiratory protective equipment (masks, several types of Filtering Facepiece Respirators - FFRs) affected daily life, and sport. FFRs are used in many professionsoften for long durations and without the possibility of removal to prevent contamination [10]. Although FFRs reduce the risk of infection, they also create measurable inspiratory resistance, which has been associated with subjective discomfort and altered ventilatory control in healthy subjects [11]. Experimental studies applying inspiratory loads—analogous to respirator use—demonstrate significant changes in tidal volume, inspiratory time, and respiratory muscle activation [12]. In some cases, there were even running competitions with mandatory mask wearing, which athletes had to wear between performances [9]. Potentially negative effects of long-term FFR wear include communication difficulties [13], increased concentration of inhaled CO_2_, hypercapnia [14,15], hypoxemia [16], headache [17], decrease in V_E_ (minute ventilation) and VO_2_max during exercise [18], skin reactions including acne [19], as well as psychological symptoms including fatigue and sleepiness [17]. These symptoms can be caused by decreased oxygen saturation during several hours of FFR wear [20] together with increased reabsorption concentration of CO_2_ [21]. Deadspace within the FFR can lead to a seven-fold increase in reinhalation of CO_2_ [22,23].

Other studies [24,25] describe the negative effect of FFRs on lung function, such as a decrease in forced vital capacity. Changes in both inspiratory and expiratory values have been noted depending on the intensity of physical activity [26,27]. Face coverings resist both the inhale and the exhale, resulting in higher activation of the respiratory muscles and impaired ventilation at high intensity [18,24]. In addition, they can also affect microclimatic conditions (temperature and humidity) in the mouth and nose regions, which reduces the cooling effect of the mucous membrane and thus may alter the subjective perception of breathing freedom [23].

Specific investigations with FFP2/N95 masks have further reported impaired ventilation at high exercise intensity and increased work of breathing [24,27]. According to Darnell et al. [9], repeated graded exercise test (GXT) results in a reduction of exercise time of 7–14% when wearing masks or respirators; Driver et al. [18] reports a decrease of VO_2_max up to 29%. Caretti & Whitley [28] showed that an inspiratory resistance ≥0.39 kPa led to a significant reduction in time to exhaustion due to a decrease in V_E_ and an increased ventilatory equivalent of oxygen (V_E_·VO_2_^−1^). Lott et al. [29] found that fabric masks reduced VO_2_max and increased time to exhaustion, suggesting a link between air flow and metabolic stress. In contrast, Epstein et al. [30] showed no significant changes in heart rate, respiratory rate, blood pressure or SpO_2_ saturation. Kyung et al. [25] reported that COPD patients experienced increased respiratory rate, respiratory alkalosis and hypocapnia when wearing an N95 respirator. While the additional airflow resistance imposed by FFR is accompanied by myriad changes in respiration, it is not yet clear if it changes the coordination of ribcage compartments associated with breathing.

There are several diagnostic methods for breathing pattern analysis [31,32], with opto-electronic plethysmography (OEP) considered to be the gold standard in the indirect evaluation of respiratory muscle work. It uses kinematic analysis of individual chest compartments, to provide indirect information on respiratory muscle involvement [33–37]. This method uses a network of 89 reflective markers and eight infrared cameras to quantify volume changes in different chest wall compartments such as the pulmonary ribcage (RCp), abdominal ribcage (RCa), and abdomen (Ab) [38].

A detailed assessment of the thoracoabdominal breathing pattern is essential because global ventilatory parameters alone do not reveal how the work of breathing is distributed across respiratory muscle groups. The relative contributions of the pulmonary ribcage, abdominal ribcage and abdomen reflect diaphragmatic function, mechanical efficiency and the ability to maintain coordinated chest wall motion during exercise [39]. Altered compartmental involvement has been linked to increased work of breathing, reduced ventilatory efficiency and earlier respiratory muscle fatigue, all of which may impair endurance performance [40]. In athletes, suboptimal thoracoabdominal coordination can limit tidal volume expansion and contribute to paradoxical ribcage motion at higher intensities [41]. Because FFRs impose additional inspiratory resistance, they may further challenge chest wall coordination and shift breathing toward less efficient patterns [26]. Therefore, thoracoabdominal kinematics provide mechanistic insight beyond conventional ventilatory metrics and are crucial for understanding how FFR use may influence breathing strategy in competitive runners.

Published studies of FFR use during exercise have focused mainly on global ventilation parameters such as respiratory rate or VO_2_max, while changes in the involvement of individual compartments during exercise have been investigated only to a limited extent [31,38,42,43]. Therefore, the aim of this study was therefore to determine whether the use of FFR influences the respiratory pattern and relative engagement of individual breath compartments in competitive runners under load, using OEP. Moreover, a partial aim of the study was to focus on intergender differences.

## 2. Research design and methods

### 2.1. Participants

Twenty middle- and long-distance runners (10 males and 10 females) from the South Bohemia region volunteered to participate in the study. A total of 21 runners were approached, but one refused to participate. All participants provided written informed consent prior to enrollment. Inclusion criteria were: (1) competitive running experience for at least 3 years, (2) a training frequency of ≥6 sessions per week with a minimum training volume of ≥35 km·week^−1^, and (3) age between 16 and 25 years. The runners were recruited through local athletics clubs in the South Bohemia region. Coaches were contacted and informed about the study, and eligible athletes were subsequently invited to participate on a voluntary basis. Exclusion criteria included smoking, musculoskeletal injury, chronic or acute respiratory diseases, and any contraindications to maximal exercise testing. The participants’ anthropometric parameters and VO_2_max values were measured through the Cardiopulmonary exercise test two weeks before the first testing. We received information from the athletes and their coaches about their training volume over the past year (see Table 2). Because the set of participants was homogeneous, we assumed it was without subgroups of athletes who could distort the results.

### 2.2. Ethics Statement and Study Registration

The research was carried out with the consent of the Ethics Committee, Faculty of Education, University of South Bohemia, Ref. No.: EK031/2023. All procedures performed in the study were in accordance with the ethical standards of the institutional research committee and all procedures adhered to the Declaration of Helsinki. Prior to the measurements, all participants or their parents signed an informed consent form. They were recruited between 15 and 30 January 2025. Trial registration: ClinicalTrials.gov number: NCT07032740. This study was registered only after participant recruitment had begun. The initial phase of the project was designed as an exploratory methodological investigation, and trial registration was completed later to ensure transparency once the intention to publish the study emerged. Ethical approval had been granted prior to data collection, and all procedures followed institutional and international guidelines. The authors confirm that all ongoing and related trials for this intervention are registered.

### 2.3. Measures, design and procedures

#### 2.3.1. Study design.

**Fig 1:**
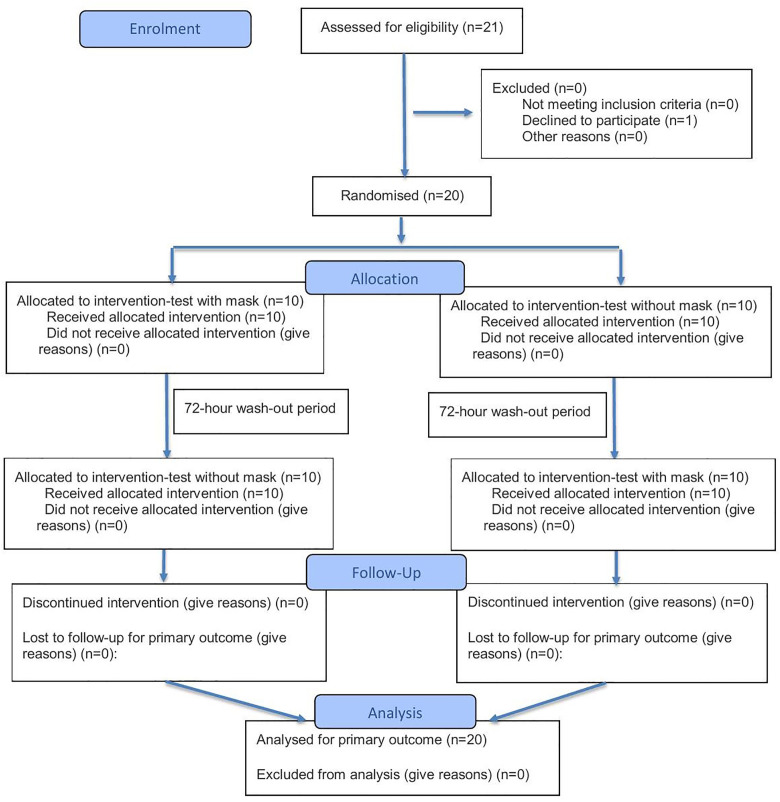
CONSORT 2025 Flow Diagram of the study with crossover design.

This clinical study employed a randomized, controlled, crossover design (see Fig 1), in which each participant completed two graded exercise tests (GXT): one performed while wearing an N95 filtering facepiece respirator (FFR) and one performed without a respirator (control condition). The order of conditions was randomized and counterbalanced using a computer-generated sequence (Randomizer.org) by the principal investigator. The tests were separated by 72 hours to minimize residual fatigue. All measurements were conducted by experienced laboratory staff who had received standardized training. Outcome assessors and data analysts, with the exception of the principal investigator and the tester, were blinded to the condition assignment throughout the study, blinding was maintained until all measurements were completed and the dataset was fully prepared for statistical analysis.

#### 2.3.2. Respirator specification.

The N95 respirator used in this study (Promedor24, Czech Republic) provides ≥95% filtration efficiency for 0.3 μm particles as the gold standard of protection against aerosol transfer during the COVID-19 pandemic [44]. The inspiratory resistance is ≤ 35 mmH_2_O at an airflow of 85 L·min^−1^, corresponding to typical ventilatory flow during heavy exercise. The internal dead-space volume ranges from approximately 130–170 cm³, depending on individual facial morphology. It is certified under the European EN 149:2001 + A1:2009 standard for FFP2 respirators, with a declared filtration efficiency of ≥ 95%.

#### 2.3.3. Graded exercise test (GXT).

The involvement of the individual breathing compartments was measured in sitting and standing positions and at each stage of the load test. For GXT, the Bruce protocol (see Table 1) was used on a treadmill (Lode Valiant 2 Sport, Lode B.V., Groningen, Netherlands). Each stage lasted 3 minutes each, and the data at the 3rd minute in each stage were evaluated. Measurements in each phase took 60 seconds. For each participant, the tests were performed at the same time of day. The test was terminated by the subject at the moment of subjective exhaustion.

**Table 1:** Bruce Protocol Stage Design – speed and incline parameters for each stage of the Bruce treadmill protocol.

Stage	Speed (km·h^−1^)	Incline (%)
1	2.7	10
2	4.0	12
3	5.4	14
4	6.7	16
5	8.0	18
6	8.8	20

#### 2.3.4 Optoelectronic plethysmography (OEP).

**Fig 2:**
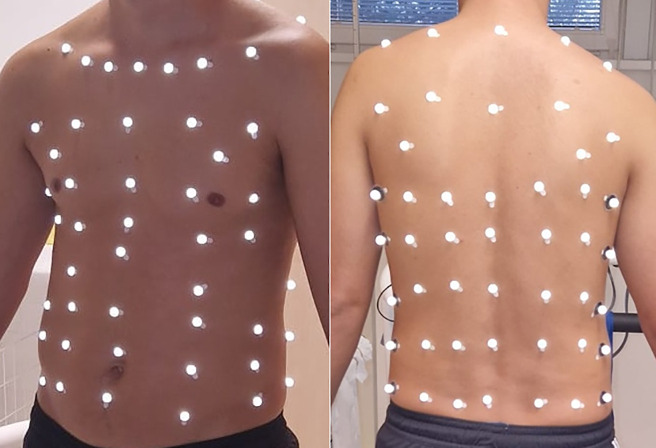
The markers set up from front and back.

An optoelectronic plethysmograph (BTS Bioengineering, Milan, Italy) was used to detect the breathing pattern and the relative involvement of individual breathing compartments. This device is the gold standard for breathing pattern measurements in breathing at rest and under stress with sampling frequency: 60 Hz [34,43]. For analysis 89 markers were used, which were located on the chest, abdomen and back to the standard three-compartment model (RCp, RCa, Ab) (see Fig 2). These markers are continuously recorded via 8 cameras, five of which are anterior and three posterior. Dynamic calibration was performed before each trial. Raw 3D trajectories were reconstructed using BTS Smart Analyzer. Missing marker gaps shorter than 10 frames were interpolated. The data were then filtered using a 4th order low pass Butterworth filter at 6 Hz. Volumes of the RCp, RCa, and Ab compartments were computed using the Gauss divergence method.

**Fig 3:**
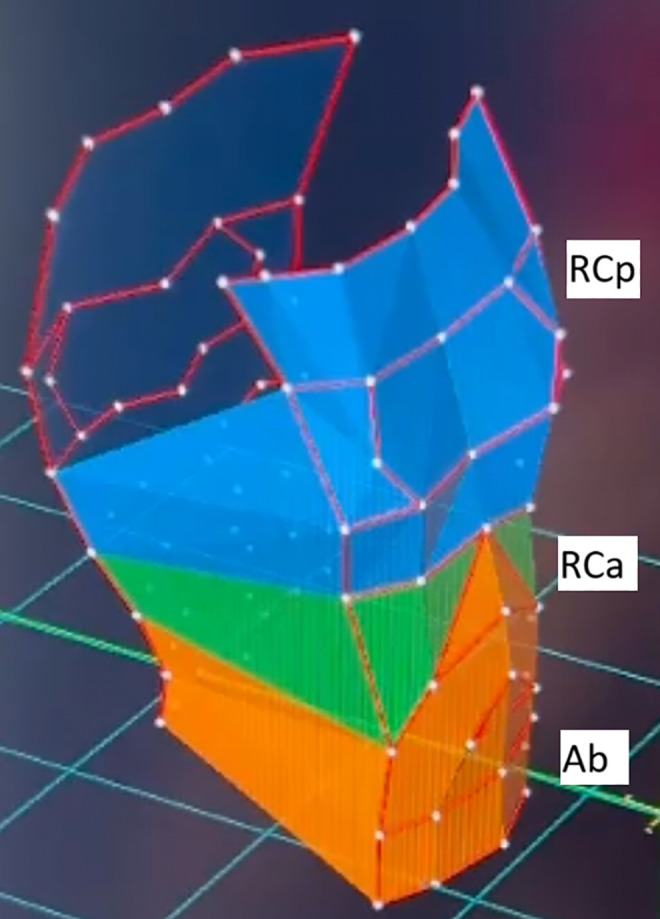
Breathing compartment model used for analysis.

The relative contribution of these three compartments to tidal volume (V_T_) (%) was compared: RCp - pulmonary rib cage, RCa - abdominal rib cage, Ab – abdomen (see Fig 3). Their involvement was determined using the difference between end-inspiratory and end-expiratory volume. Parameters were also measured: V_T_, B_F_ (breathing frequency), V_E_.

### 2.4. Statistical analysis

Data were anlyzed using linear mixed-effect model (LM) [45] with the formula:

$Y_{ij}=\beta_{0}+\beta_{1}Gender_{j}+\beta_{2}Phase_{ij}+\beta_{3}Respirator_{ij}+\beta_{4}Phase_{ij}\times Respirator_{ij}+\beta_{5}Period_{ij}+\beta_{6}Sequence_{j}+u_{0j}+\epsilon_{ij}$

Here, **Gender** and **Phase** represent standard fixed effects. The term **Phase** × **Respirator** denotes the fixed interaction effect, which allows an estimate of the difference associated with using the N95 respirator separately for each phase. The term $u_{\left\{\text{0}j\right\}}$ represents the random intercept for each participant *j*; this accounts for the correlation between repeated measurements gathered from the same individual (i.e., measurements taken with and without the N95 respirator across different phases), remains retained for modelling the intra-subject correlation. The β coefficients denote the model parameters corresponding to each effect, and $\epsilon_{\left\{ij\right\}}$ represents the residual error. The *Period*_*ij*_ is a categorical variable (1st vs. 2nd test day). It allows us to filter out any systematic change in thoracic abdominal volumes that occurred between visits. Finally *Sequence*_*j*_ is a categorical variable identifying a group (Mask → No-Mask vs. No-Mask → Mask). This term is detecting the presence of the order effect.

The models were computed in software R using the package lme4 [45].

Data were presented as mean and standard deviation. Since 268 values enter the overall analysis, the central limit theorem supports the analysis of the data using LM, since it is robust to nonnormality in cases of a large amount of data. Significance was set at the α = 0.05 level. Since all predictors were categorical, the unstandardized estimates (Estimate values) from the model can be directly interpreted as effect sizes, representing the actual magnitude of change in respiratory compartment involvement. These values were thus used to assess practical (clinical) significance of the observed effects without the need for further standardization. Changes reaching or exceeding 2–4 percentage points were considered practically meaningful based on previously reported physiological thresholds in performance-related respiratory adaptations [6]. Data processing was performed using R software, version 4.3.1 (R Foundation for Statistical Computing, Vienna, Austria).

## 3. Results

Table 2 provides an overview of the anthropometric characteristics, aerobic capacity (VO_2_max), and weekly training volume for male and female participants.

**Table 2:** Anthropometric characteristics, aerobic capacity, and training volume of male and female participants.

	age (years)	mass (kg)	height (cm)	VO_2_max (mL·kg^−1^·min^−1^)	training volume (km/week)
males (n = 10)	19.04 ± 2.02	71.35 ± 9.08	184.43 ± 5.71	60.96 ± 4.02	60.2 ± 6.7
Females (n = 10)	18.69 ± 2.48	58.37 ± 3.45	170.31 ± 3.86	54.69 ± 3.96	52.5 ± 10.1

Some participants achieved a different final stage in the GXT (Bruce protocol) test – see Table 3. The distribution of the final completed stage during the Bruce protocol among male and female participants is presented. The FFR affected the length of the test: males had a reduction in the length of the test by 79 ± 61 seconds, and females by 25 ± 27 seconds.

**Table 3:** Participant Final Stage Reached in Bruce Test.

Final Stage (Bruce Protocol)	12th min	15th min	18th min
Number of males (n)	2	5	3
Number of females (n)	7	3	0

**Fig 4:**
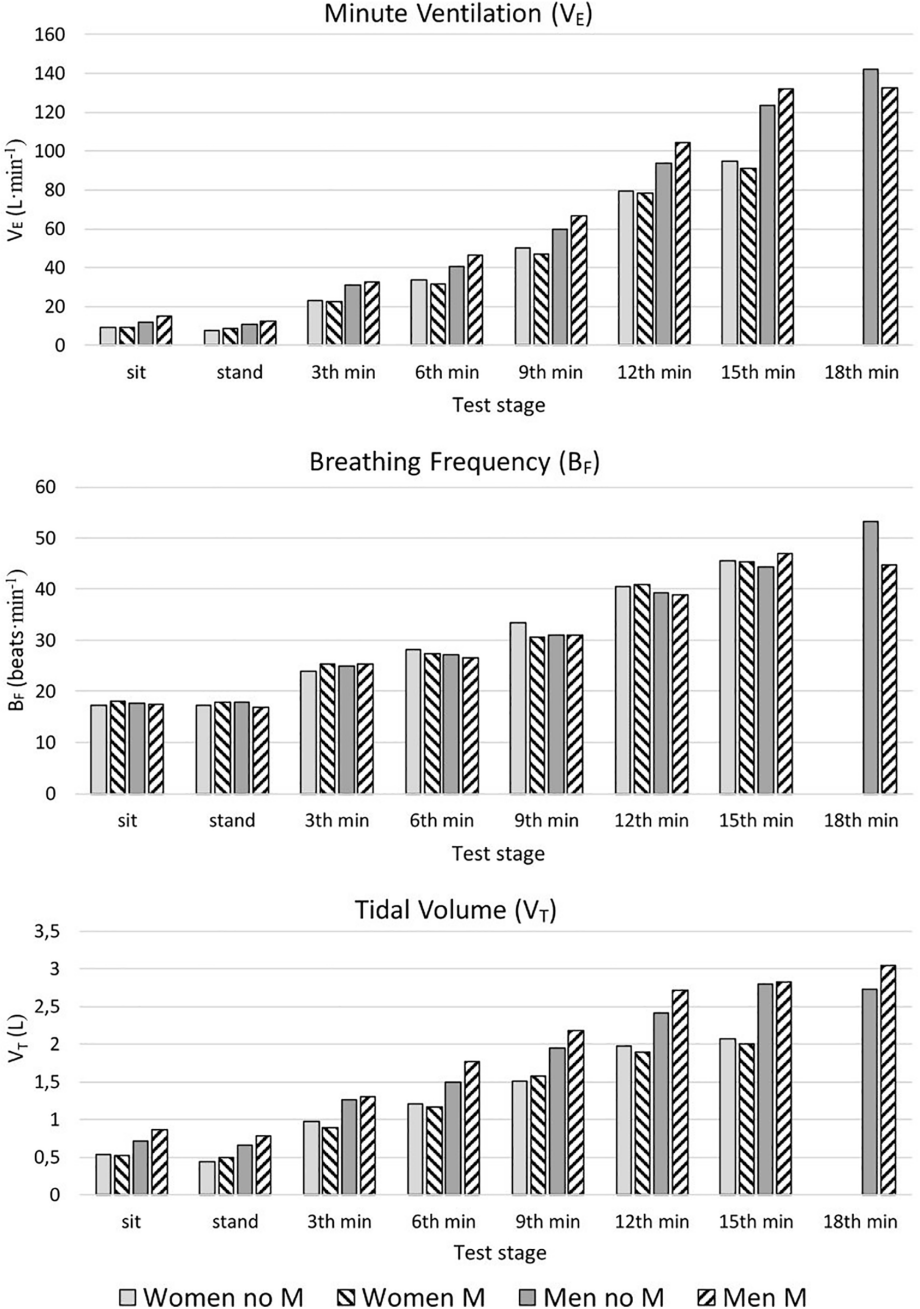
Values of V_T_, B_F_ and V_E_ in FFR and non-FFR test in males and females. Notes: no M – unmasked, M – masked.

There were no significant effects of FFR, gender, or phase (sitting, standing, or GXT) upon V_T_, B_F_ or V_E_ (Fig 4). V_T_ was generally higher for masked males under load and for all phases except the 18th minute for V_E_, but the differences were not statistically significant.

**Fig 5:**
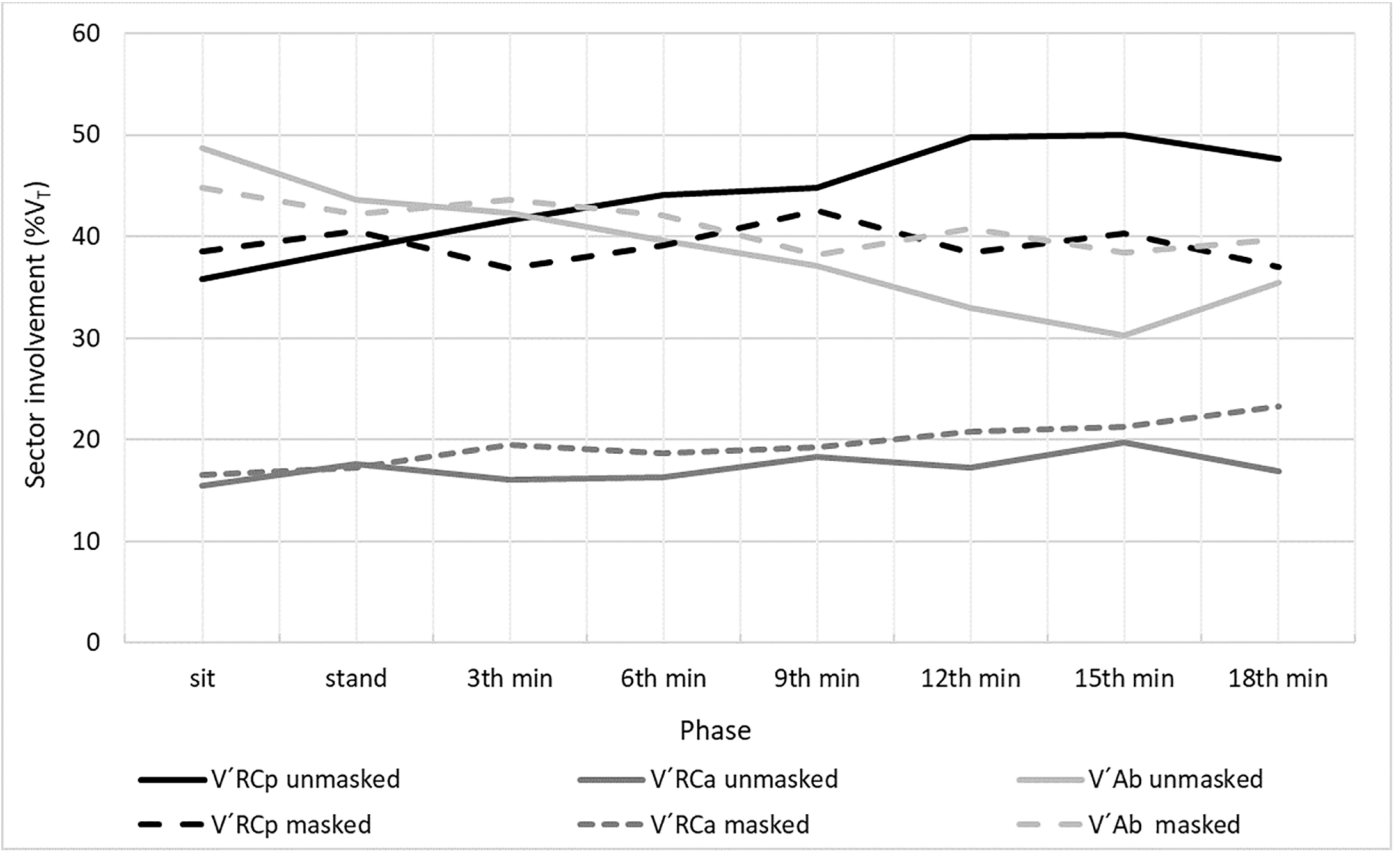
The contribution of individual breathing compartments during sitting, standing and every 3rd minute of six stages of GXT in male runners. Notes: V´RCp - pulmonary rib cage volume, V´RCa - abdominal rib cage volume, V´Ab – abdomen volume.

**Fig 6:**
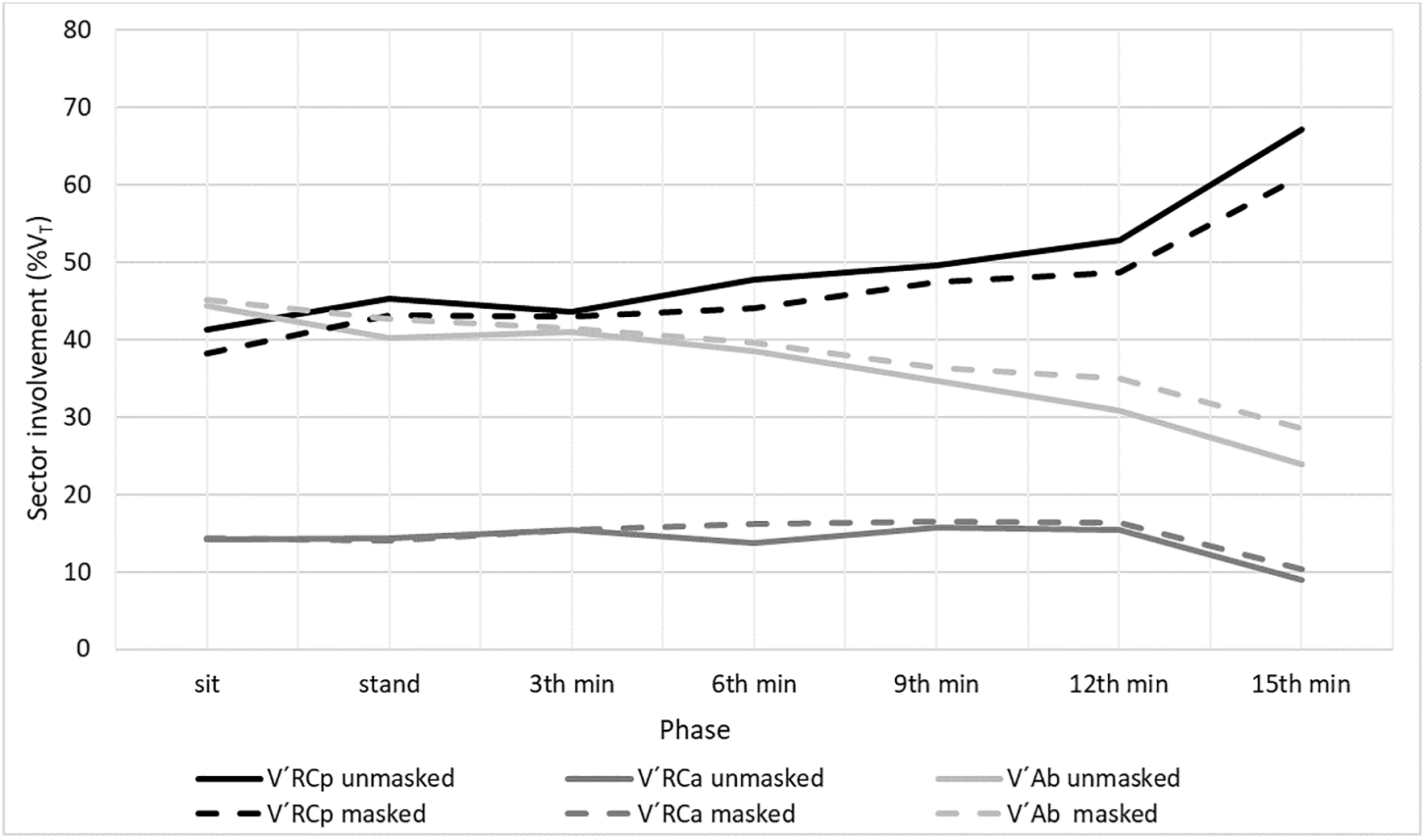
The contribution of individual breathing compartments during sitting, standing and every 3rd minute of six stages of GXT in female runners. Notes: V´RCp - pulmonary rib cage volume, V´RCa - abdominal rib cage volume, V´Ab – abdomen volume.

The contribution of the Ab and RCa compartments increased s and the contribution of the RCp compartment decreased in both males and females with FFR (see Figs 5 and 6).

**Fig 7:**
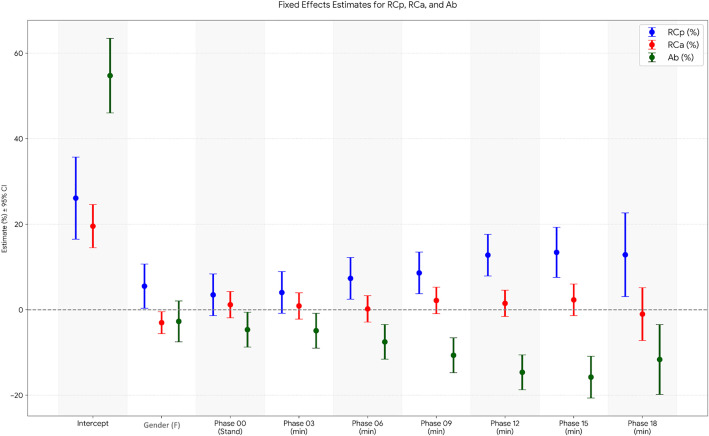
Main effects estimate for RCp, RCa and Ab together with their 95% confidence intervals.

As shown in Fig 7, the average breath compartment involvement in breath intercept data was: 36.0: 16.2: 47.8% (RCp: RCa: Ab). The intercept data represent the values of males, in the sitting position, without FFR. Females have a different breath compartment involvement than males, with females having a greater contribution to the RCp compartment at the expense of the RCa and Ab compartments. Across all phases, females demonstrated greater RCp dominance (P = 0.052) compared to males. The involvement of individual breath compartments differs in each monitored phase from sitting (phase03.min to phase18.min of Fig 7). There was a significant increase in the proportion of the RCp compartment from the 6th minute of the test to the end of the test and a significant decrease in the proportion of the Ab compartment in all phases of the test, also while standing. See the deviation of 95% confidence intervals from zero in Fig 7.

**Fig 8:**
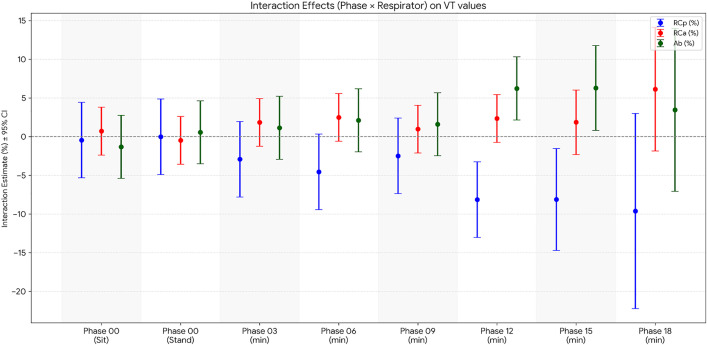
Interaction Effects (Phase × Respirator) on V_T_ values for individual compartments together with their 95% confidence intervals.

In addition to statistical significance, the estimated effects suggest physiologically relevant shifts in thoracoabdominal breathing patterns. For instance, the interaction between phase and respirator at the 12th minute led to a reduction of RCp contribution (see Fig 8) by approximately 7.85 percentage points (P = 0.002), accompanied by a compensatory increase in abdominal contribution (Estimate = +5.97, P = 0.004). These changes exceed 20% relative to baseline values for each compartment, indicating not only statistically significant but also practically meaningful adaptations in breathing strategy under load.

The analysis showed that the period effect was significant for RCp and Ab. Specifically, RCp was on average 2.97 units higher in the first measurement compared to the second, whereas Ab was 2.53 units lower in the first measurement than in the second. In contrast, the period effect for RCa was not statistically significant. Regarding the sequence effect, we also tested for its potential influence. The results showed that the sequence effect was not statistically significant for any of the examined variables, indicating that the order in which participants underwent the testing protocol did not meaningfully affect the outcomes.

The period effect, which can arise when the exercisers are already tired or have adapted better to the test, or there have been some physiological changes between measurements was detected to be significant for RCp and Ab. RCp is systematically 2.97 larger in the first measurement than in the second, while Ab is 2.53 smaller in the first measurement than in the second. RCa came out insignificantly. The sequence effect was statistically not significant, indicating that the order of testing did not influence the measured outcomes.

## 4. Discussion

The present study examined the effects of N95 respirator use on compartment-specific thoracoabdominal breathing mechanics in trained runners during graded treadmill exercise with optoelectronic plethysmography (OEP). While performance was reduced, the core ventilatory parameters–V_T_, B_F_, and V_E_–remained unaffected by the FFR, consistent with prior findings in healthy individuals during short-term mask use [38,46]. These findings provide new insight into the respiratory strategies employed during inspiratory loading and contribute to the growing body of literature evaluating the physiological effects of facepiece respirators during exercise.

The key finding of this study was a significant shift in the relative contributions of thoracoabdominal compartments. Specifically, there was a reduction in the rib cage pulmonary (RCp) contribution and an increase in abdominal (Ab) activity, particularly at higher intensities. While the interaction effects were most pronounced at minutes 12 and 15, statistical significance was not observed at minute 18, likely due to the reduced number of participants completing this phase. The relative engagement of Rca does not change during the different exercise intensities, which is consistent with previous findings [42]. Several physiological mechanisms may explain the decreased RCp contribution while wearing the respirator: increased inspiratory resistance imposed by the N95 may elevate the work of the external intercostal muscles, leading to earlier recruitment of abdominal muscles for volume generation [12]. Alternatively ribcage stiffening caused by elevated negative intrathoracic pressure may reduce the mobility of the upper thorax, redirecting volume expansion toward the abdomen [47]. Greater activation of the diaphragm under inspiratory load may encourage abdominal displacement as a more energetically efficient strategy at high intensities [12]. The corresponding increase in Ab contribution highlights the ability of trained athletes to redistribute mechanical work among respiratory compartments to maintain ventilation under load. Such compensation may protect against early respiratory muscle fatigue [48].

The magnitude of these changes was not only statistically significant but also practically relevant. A reduction in RCp by approximately 7–8 percentage points and a corresponding increase in Ab contribution represents a ~ 20% relative change in tidal volume distribution, given typical RCp values of 35–40% in trained runners [49]. These findings suggest a substantial redistribution of ventilatory work from the upper rib cage to the abdominal region, a known compensatory response to elevated inspiratory resistance [7,11].

Although some phase-by-respirator interactions were not statistically significant, effect sizes (e.g., 2–4 percentage points) may still carry relevance in elite athletes, where even small biomechanical changes can impact endurance, fatigue onset, or posture [8,18]. This redistribution likely reflects a high degree of respiratory plasticity and functional reserve [35] among trained runners. Conversely, the increased Ab contribution to support ventilation could compromise the postural responsibilities of the abdominal wall. This may manifest over longer duration of FFR use during exercise, but this phenomenon was not a primary aim of this study [50].

A potential sequence effect was also considered, as it could theoretically arise if participants first performed the test with a respirator and then without it, or vice versa. Under such circumstances, a systematic shift in one direction could occur due to the order in which the respirator was used. In our study, some participants started the protocol wearing a respirator, whereas others began without it; this allocation was purely random. The randomized assignment of starting conditions was therefore intended to prevent any systematic bias related to testing order. To verify this statistically, we additionally included the sequence effect in the model, and the analysis confirmed that it was not significant. We also examined the period effect, which may appear when participants are already fatigued, have better adapted to the test procedure, or if physiological changes occur between repeated measurements. After incorporating this effect into the model, it emerged as significant for RCp and Ab. Specifically, RCp was systematically 2.97 units higher in the first measurement than in the second, whereas Ab was 2.53 units lower in the first measurement compared to the second. In contrast, RCa showed no significant period effect. Finally, we considered the possibility of a carryover effect, which may occur when the influence of a previous test persists and affects subsequent measurements in cases where the time interval between tests is insufficient. Given the structure of our experimental design, the carryover effect cannot be statistically separated from the period effect. Therefore, any potential carryover influence is inherently captured within the observed period effect estimates.

The size of clusters is considered “informational” if the number of observations in a given individual is directly related to the resulting value of the measured quantity. In the presented study, the size of the cluster is determined by how many phases of the Bruce Protocol the runner completed before reaching subjective exhaustion. According to Table 3 in the manuscript, the size of the clusters ranges from 4 to 6 stages (12–18 minutes).

Exercisers who completed the test earlier are likely to have less lung capacity and therefore may experience a different compensation of breathing pattern than exercisers who completed the test later. However, due to the nature of the Bruce protocol, which ran until free exhaustion, there is a variable number of observations per participant in our data (clusters of 4–6 phases). This situation requires consideration of the phenomenon of information cluster size (ICS), where the duration of the test correlates with the physiological fitness of the subject. On a theoretical level, ICS could lead to over-influencing population averages by runners with the highest endurance. In our study, however, this risk is minimized by the high homogeneity of the training of the cohort and the fact that the primary goal – the evaluation of the effect of the respirator – was implemented as an intra-subject comparison within each cluster.

Notably, gender-specific trends were observed in ventilatory responses to the use of FFR, including V_E_ and V_T_, as well as in thoracoabdominal contribution. Male participants, typically exhibiting larger lung volumes and stronger diaphragmatic function, compensated for inspiratory resistance primarily through increased V_T_ and V_E_. Female participants displayed relatively less thoracoabdominal adaptation, likely due to anatomical constraints and reduced absolute metabolic demand. These findings are consistent with established gender differences in breathing mechanics and muscle recruitment [40].

Parallel to prior findings, short-term FFR use does not substantially impair ventilation in healthy individuals [9,30], but may modify breathing mechanics. Our results expand this understanding by demonstrating the ability of runners to adapt through compartment-specific recruitment strategies, contrasting with general populations [38,51], where such adaptations are limited.

While FFRs are known to increase CO_2_ rebreathing [15] and inspiratory resistance [24], runners may offset these effects through improved diaphragmatic strength, thoracoabdominal coordination, and efficient ventilatory timing [1,2,9]. Darnell et al. [9] observed reductions in time-to-exhaustion under mask conditions despite unchanged heart rate and saturation, consistent with our observations of altered breathing strategies rather than ventilatory failure.

When compared to untrained individuals, the runners in our study displayed significant shifts in thoracoabdominal activity during exercise, particularly through enhanced Ab and RCa activation [52]. These differences are attributable to long-term training adaptations, such as increased respiratory muscle strength, enhanced proprioception, and reduced ventilatory load at a given intensity [53]. Our compartmental analysis revealed that, in males at rest and without FFR, the Ab region contributed nearly 48% to ventilation, compared to ~36% for RCp and ~16% for RCa. This pattern reflects efficient diaphragmatic breathing and aligns with prior findings on optimized respiratory mechanics in trained individuals [54]. During the change of position and during the load, there were changes in this ratio. Females consistently exhibited greater RCp contribution across phases, consistent with previous evidence indicating a more thoracic-dominant breathing pattern in women [40]. At high exercise intensities, mask use significantly increased perceived exertion in both genders, consistent with Hidaka et al. [55] and Zheng et al. [46], who observed similar trends in subjective effort.

From an applied perspective, these results suggest that although basic ventilatory parameters remain stable under FFR use, breathing mechanics are significantly altered. Coaches and sport scientists should be aware of these shifts when designing training or recovery strategies under masked conditions [53]. While acute FFR use appears safe, individualized consideration is necessary, particularly for high-performance contexts [53,56,57]. This study provides new insights into thoracoabdominal compensation strategies under inspiratory load in highly trained runners. The findings underscore the adaptability of respiratory coordination and highlight the potential for individualized respiratory assessment and training aimed at maintaining performance despite mechanical constraints.

### Limitations

Although the study provides novel findings, several limitations should be acknowledged: The sample consisted of young, trained runners, limiting generalizability to general or clinical populations. The sample size was small, particularly in the later stages of the test protocol, and therefore limits the statistical power for subgroup analyses, including sex‑specific effects. Only one respirator model (N95) was tested; results may differ with other FFR designs. OEP data were collected at discrete time points, not continuously, which may overlook transient changes. No direct measurement of respiratory muscle activation (e.g., EMG) was included, limiting mechanistic interpretation.

## 5. Conclusion

This study demonstrated that the use of the FFR (N95) does not affect basic ventilation parameters, such as V_T_, B_F_, and V_E_. But causes significant changes in the involvement of individual breath compartments during GXT in race runners. The results also showed gender differences and the effect of postural and load changes on the breathing pattern. Race runners exhibit sophisticated compensatory mechanisms that allow respiratory efficiency to be maintained even with additional respiratory resistance. It has been newly demonstrated that the upper thoracic compartment is preferred with increasing stress without FFR, while the proportion of abdominal activity increases at higher stages with the use of FFR. Although the short-term use of FFR appears to be tolerable, the long-term consequences for training practice and performance remain unclear. These results highlight the potential of OEP in the evaluation of breathing patterns in athletes, which supports the further development of personalized breathing training in the context of specific sporting demands. Understanding these changes can help coaches and clinicians develop individualized respiratory training strategies to improve performance and safety under constrained breathing conditions. This has potential relevance for athletes training in polluted environments, at altitude, or during respiratory pandemics.

## Supporting information

S1 File Clinical study protocol describing the study design, participants, procedures, and statistical analysis plan. (PDF)

S2 File CONSORT 2025 checklist detailing reporting of the randomized crossover trial. (DOCX)
