# Supplementary material for: Respiratory pattern change in female and male runners by respiratory tract restriction using a respirator
Source: PLoS One. 2026 Jul 16;21(7):e0353784. doi: 10.1371/journal.pone.0353784 (PMC13374970; doi:10.1371/journal.pone.0353784)
Supplement: S1 File — (PDF) [file pone.0353784.s001.pdf]

Clinical Study Protocol

Protocol Name: Changing Respiratory Patterns in Runners by Restricting Airways Using a Respirator

Date: September 15, 2023

Principal investigator:
PhDr. Petr Bahenský, Ph.D., KTVS PF USB

Team leaders:
Mgr. David MarkoMgr. Miroslav KrajcigrDoc. PhDr. Renata Malátová, Ph.D.

Place of implementation of the study:
Laboratory of Functional Stress Diagnostics – KTVS PF USB, České Budějovice

Funding: None

1. Introduction and rationale of the study

Breathing pattern and ventilation efficiency play a key role in endurance performance. Restricting the airway with respirators (e.g., N95/FFP2) can alter breathing mechanics, ventilation responses, and breathing patterns at rest and under exercise. Understanding these influences is important for athletes, coaches and healthcare professionals. Despite extensive research during the pandemic, COVID19 still lacks detailed kinematic data of the breathing pattern using optoelectronic plethysmography (OEP) in trained endurance runners.

The aim of this study is to provide accurate physiological and kinematic data to describe how the respirator affects the breathing pattern at rest and during gradually increasing load in trained runners.

2. Goals and hypotheses

2.1 Primary Objective

To determine the effect of N95 respirator use on breathing pattern during the stress test (GXT) in trained middle and long distance runners.

2.2 Secondary objectives

- Assess the effect of the respirator on ventilation parameters (VE, VT, BF, VO2, VCO2, RER).
- Compare the relative proportion of the chest and abdominal compartments to the inspiratory volume with and without a respirator.
- To find out if gender modifies the respiratory response to respirator use.

2.3 Hypotheses

- H1: Using a respirator will increase your respiratory rate and decrease your tidal volume during GXT.

- H2: The use of a respirator will lead to greater involvement of the abdominal compartment.
- H3: Female runners will show different sector engagement than male runners.

3. Design study

This is a randomized controlled crossover trial with two conditions:

- **GXT with N95 respirator**
- **GXT without respirator**

Each participant will take both tests in random order, at least 72 hours apart.

3.1 Randomization

A random sequence will be generated using Randomizer.org. The allocation is done by the principal investigator. Assessors and analysts will be blinded to assign conditions.

3.2 Course of the study

- Visit 1: Informed consent, input data, familiarization with the procedure, GXT (condition A)
- Visit 2 (≥72 hours): GXT (condition B)

When finished, the CONSORT diagram will be added.

4. Participants

4.1 Inclusion criteria

- Age 16-25 years
- Medium and long distance runners
- At least 3 years of racing experience
- ≥6 workouts per week
- ≥35 km per week

4.2 Exclusion criteria

- Smoking
- Musculoskeletal injuries
- Acute or chronic respiratory disease
- Contraindications to maximum load

4.3 Recruitment

Participants will be recruited through running clubs, coaches, and university channels. A total of about 20 people (10 women, 10 men) will be included.

5. Interventions and procedures

5.1 Pre-Test Standardization

Participants will be instructed to:

- avoid intense exercise 24 hours before the test
- follow a similar diet and hydration regimen
- Do not take caffeine 4 hours before the test
- take both tests at the same time of day

5.2 Condition with a respirator

Respirator: N95 (Promedor24, Czech Republic), efficiency ≥95%.

5.3 Stress Test (GXT)

- Bruce Protocol on the Treadmill (Lode Valiant 2 Sport)
- Each stage lasts 3 minutes
- Analysis of data from the last minute of each stage
- Test completed at subjective exhaustion

5.4 Breathing Pattern Measurement (OEP)

System: Optoelectronic Plethysmography (BTS Bioengineering, Italy)

- 89 markers on the chest, abdomen and back
- Sensing: 60 Hz
- 8 cameras (5 front, 3 rear)

Data processing:

- Reconstruction of 3D trajectories (BTS Smart Analyzer)
- Interpolation of missing data
- 4th order Butterworth filtration, 6 Hz
- Calculation of volumes RCp, RCa, Ab by Gaussian divergence method
- Calculation of relative contributions (%) for each breath cycle

6. Rated indicators

6.1 Primary Indicator

- Relative Contribution of RCp, RCa, and Ab Sections to Tidal Volume During GXT

6.2 Secondary indicators

- Ventilation parameters (VE, VT, BF, VO2, VCO2, RER)
- Heart rate
- Perceived exertion (Borg scale)

7. Statistical plan

7.1 General approach

- Normalita posouzena Shapiro–Wilkovým testem.
- Pair comparison: ANOVA with repeated measurements or linear mixed models (condition × degree × sex).
- In the case of abnormality, nonparametric alternatives are used.
- Correction for multiple testing: Bonferroni.
- Significance level $\alpha = 0.05$.
- Software: R (version 4.x) and JASP.

7.2 Missing data

- Unique thanks to controlled conditions
- Short spaces in OEP interpolated
- Incomplete GXT → exclusion from specific analyses

8. Management that

- Personal data stored separately on a secure computer.
- Anonymization within 3 days after testing.
- Only the principal investigator has access to identifiable data.
- Data stored for at least 10 years.

9. Ethical aspects

- Written informed consent will be obtained prior to the start of the study.
- For minors, the consent of a legal guardian is required.
- Participants can withdraw at any time.
- The study complies with the ethical standards of the Faculty of Law USB.
- No photos or videos will be taken.

10. Risks and precautions

- The test may cause fatigue, dizziness or discomfort.
- Immediately interrupt the test by raising your hand.
- Testing is carried out by experienced staff.
- Equipment calibrated regularly.
- The presence of a trained person and a first aid kit.
