# Supplementary material for: Respiratory pattern change in female and male runners by respiratory tract restriction using a respirator
Source: PLoS One. 2026 Jul 16;21(7):e0353784. doi: 10.1371/journal.pone.0353784 (PMC13374970; doi:10.1371/journal.pone.0353784)
Supplement: Attachment — Submitted filename: Response to Reviewers.docx [file pone.0353784.s004.docx]

PONE-D-25-61828

Respiratory pattern change in female and male runners by respiratory tract restriction using a respirator

Journal Requirements:

When submitting your revision, we need you to address these additional requirements.

1. Please ensure that your manuscript meets PLOS ONE's style requirements, including those for file naming. The PLOS ONE style templates can be found at

https://journals.plos.org/plosone/s/file?id=wjVg/PLOSOne_formatting_sample_main_body.pdf and

https://journals.plos.org/plosone/s/file?id=ba62/PLOSOne_formatting_sample_title_authors_affiliations.pdf

**I checked**

2. We note that you have selected “Clinical Trial” as your article type. PLOS ONE requires that all clinical trials are registered in an appropriate registry (the WHO list of approved registries is at https://www.who.int/clinical-trials-registry-platform/network/primary-registries" https://www.who.int/clinical-trials-registry-platform/network/primary-registries and more information on trial registration is at http://www.icmje.org/about-icmje/faqs/clinical-trials-registration/). Please state the name of the registry and the registration number (e.g. ISRCTN or ClinicalTrials.gov) in the submission data and on the title page of your manuscript. a) Please provide the complete date range for participant recruitment and follow-up in the methods section of your manuscript. b) If you have not yet registered your trial in an appropriate registry, we now require you to do so and will need confirmation of the trial registry number before we can pass your paper to the next stage of review. Please include in the Methods section of your paper your reasons for not registering this study before enrolment of participants started. Please confirm that all related trials are registered by stating: “The authors confirm that all ongoing and related trials for this drug/intervention are registered”. Please see http://journals.plos.org/plosone/s/submission-guidelines#loc-clinical-trials for our policies on clinical trials.

**The study is registered, the necessary information is given on the first page of the manuscript as well as in the Methods section, chapter 2.2.**

3. We note that the original protocol that you have uploaded as a Supporting Information file contains an institutional logo. As this logo is likely copyrighted, we ask that you please remove it from this file and upload an updated version upon resubmission.

**We uploaded forms without the university logo into the system.**

4. If the reviewer comments include a recommendation to cite specific previously published works, please review and evaluate these publications to determine whether they are relevant and should be cited. There is no requirement to cite these works unless the editor has indicated otherwise.

**Thank you.**

Reviewers' comments:

1. Is the manuscript technically sound, and do the data support the conclusions?

The manuscript must describe a technically sound piece of scientific research with data that supports the conclusions. Experiments must have been conducted rigorously, with appropriate controls, replication, and sample sizes. The conclusions must be drawn appropriately based on the data presented.

Reviewer #1: Yes

2. Has the statistical analysis been performed appropriately and rigorously?

Reviewer #1: Yes

3. Have the authors made all data underlying the findings in their manuscript fully available?

The PLOS Data policy requires authors to make all data underlying the findings described in their manuscript fully available without restriction, with rare exception (please refer to the Data Availability Statement in the manuscript PDF file). The data should be provided as part of the manuscript or its supporting information, or deposited to a public repository. For example, in addition to summary statistics, the data points behind means, medians and variance measures should be available. If there are restrictions on publicly sharing data—e.g. participant privacy or use of data from a third party—those must be specified.

Reviewer #1: No

**The data is available on the website: http://www.petrbahensky.wz.cz/pdf/Conference010.pdf**

**This information is provided in the article in Chapter 2.6**

4. Is the manuscript presented in an intelligible fashion and written in standard English?

PLOS ONE does not copyedit accepted manuscripts, so the language in submitted articles must be clear, correct, and unambiguous. Any typographical or grammatical errors should be corrected at revision, so please note any specific errors here.

Reviewer #1: No

**One of the authors is a native speaker, he checked and edited the text.**

5. Review Comments to the Author

Please use the space provided to explain your answers to the questions above. You may also include additional comments for the author, including concerns about dual publication, research ethics, or publication ethics. (Please upload your review as an attachment if it exceeds 20,000 characters)

Reviewer #1: THis paper looks at the effect of wearing an N95 mask during exercise on various respiratory parameters. Overall, grammar and phrasing need improvement.

**Thank you for your comment, we have edited the text, one of the authors is a native speaker.**

1. In what setting were the runners recruited?

**Thank you for your comment. The runners were recruited through local athletics clubs in the South Bohemia region. Coaches were contacted and informed about the study, and eligible athletes were subsequently invited to participate on a voluntary basis.**

2. Please move table 1 and its description to the results section. In the methods section, please describe how this information was collected.

**Thank you for your comment, I moved table 1 to the results section (it is currently table 2) and in the methodology section I described the data collection.**

3. Your flow diagram makes no sense. For the first split, show how many were randomized to "mask" then "no mask" and how many to "no mask" then "mask".

**Thank you for your comment. Yes, really the diagram was not correct. We created a new one, which we inserted into the text.**

4. Please use standard formulation to show the formula of the model, and not R code.

**Thank you for your comment.** **We have edited the formula and commentary. The paragraph was rewritten in the following way:**

**To analyze the data was performed the linear mixed-effect model (LM) [42] with the formula:**

**RCp_{ij} = β_0+ β_1 (Sex_j )+ β_2 (Phase_{ij} )+ β_3 (Phase_{ij} ×Respirator_{ij} )+ u_{0j} + ϵ_{ij}**

**Here, Sex and Phase represent standard fixed effects. The term Phase × Respirator denotes the fixed interaction effect, which allows us to estimate the difference associated with using the N95 respirator separately for each phase. The term u_{0j} represents the random intercept for every person j; this accounts for the correlation between repeated measurements gathered from the same individual (i.e., measurements taken with and without the N95 respirator across different phases). Finally, the β coefficients denote the model parameters corresponding to each effect, and ϵ_{ij} represents the residual error.**

5. The language in lines 145-149 needs improvement.

**Thank you for your comment. See above point. The text was rewritten by one of the authors - a native speaker.**

6. "Finally, person is a random intercept model that allows us to model the correlation between data

148 gathered from the same person, i.e., the measurements of the same person with and without N95 respirator and in different

149 phases." Instead, "the model included a random intercept to model the correlation between observations from the same person."

**Thank you for your comment. We rewrote this sentence a bit differently due to the rewriting of the formula.**

7.Please remove the post-hoc sample size calculation. It provides no information beyond statistical significance.

**Thank you for your comment. Point 2.5 has been deleted.**

8. Instead, please describe how the initial sample size was arrived at.

**Thank you for your comment. In order to obtain credible results, we worked with a very homogeneous group of participants (i.e., top athletes, of a particular age), therefore we do not expect any subgroups of athletes in the data base that could distort the results, and therefore our data sample contains only 20 participants,** **all who met the criteria. We stated this in paragraph 2.1.**

9. You do not describe the potential for sequence effects, period effects or carryover effects. Please add methods and results to address each of these.

**Thank you for your comment. The order of the tests was randomized, with half of the participants taking the first test with a respirator and half of the participants taking the test without a respirator. We have added to the text in chapter 2.3.1.**

11. Table 4 and 5 are hard to understand. Instead, show the RCP estimates in masked and non-masked situations with 95% CIs (no t-test value, no p value). Also present the p value for the interaction (the p value from Table 5). Probably better to show as a figure.

**Thank you for your comment. We summarize the results according your advice in the following figures. We have replaced the tables with these figures.**

12. Please consider the possibility of modeling phase as a linear term?

**Thank you for your comment. Our goal was to determine how the breath pattern changes, particularly when the behavior changes, and what changes occurred in the different phases. We believe that converting the phase to a linear trend would simplify the problem and introduce the assumption of linearity, which seems defensible for the first phases but not for the later phases, when the patterning appears to have already stabilized. We would find out how the pattern changes assuming linearity, but we wouldn't find out when the pattern is already different.**

13.Please discuss the possibility of informative cluster sizes on your analysis and the potential effects on the results observed.

**See the point 8 - Thank you for your comment. In order to obtain credible results, we worked with a very homogeneous group of participants (i.e., top athletes, of a particular age), therefore we do not expect any subgroups of athletes in the data base that could distort the results, and therefore our data sample contains only 20 participants.**

14. In the discussion you say "sex relateddifferences were observed in ventiliatory responses...." To assess this properly, you need to include interactions with sex.

**Thank you for your comment. Originally, we did not assume that the respirator would have a significantly different effect on men and women. Nevertheless, we included the intersex differences in the analysis: Figure 4, Figure 7 (originally Table 4). But we modified the statement in the discussion to: „Notably observed, sex-specific trends were observed in ventilatory responses…“**
