# Supplementary material for: Respiratory pattern change in female and male runners by respiratory tract restriction using a respirator
Source: PLoS One. 2026 Jul 16;21(7):e0353784. doi: 10.1371/journal.pone.0353784 (PMC13374970; doi:10.1371/journal.pone.0353784)
Supplement: Attachment — Submitted filename: Response to review last.docx [file pone.0353784.s005.docx]

PONE-D-25-61828R1

Respiratory pattern change in female and male runners by respiratory tract restriction using a respirator

PLOS One

Review Comments to the Author

Please use the space provided to explain your answers to the questions above. You may also include additional comments for the author, including concerns about dual publication, research ethics, or publication ethics. (Please upload your review as an attachment if it exceeds 20,000 characters)

Reviewer #1: 1. Your response does not address this comment: You do not describe the potential for sequence effects, period effects or carryover effects. Please add methods and results to address each of these.

Response: We apologize to the reviewer that we were not specific considering some of his/her comment.

We incorporated the sequance effect, period effect and carryover effect into the modeling. The procedure and results are in the article on pages 16-17.

2. Your response does not adddress this comment: Please discuss the possibility of informative cluster sizes on your analysis and the potential effects on the results observed.

Response: We apologize to the reviewer that we were not specific considering some of his/her comment.

The size of clusters is considered "informational" if the number of observations in a given individual is directly related to the resulting value of the measured quantity. In the presented study, the size of the cluster is determined by how many phases of the Bruce Protocol the runner completed before reaching subjective exhaustion. According to Table 3 in the manuscript, the size of the clusters ranges from 4 to 6 stages (12 to 18 minutes). Exercisers who completed the test earlier are likely to have less lung capacity and therefore may experience a different compensation of breathing pattern than exercisers who completed the test later. However, due to the nature of the Bruce protocol, which ran until free exhaustion, there is a variable number of observations per participant in our data (clusters of 4 to 6 phases). This situation requires consideration of the phenomenon **of information cluster size (ICS),** where the duration of the test correlates with the physiological fitness of the subject. On a theoretical level, ICS could lead to over-influencing population averages by runners with the highest endurance. In our study, however, this risk is minimized by the high homogeneity of the training of the cohort and the fact that the primary goal – the evaluation of the effect of the respirator – was implemented as an intra-subject comparison within each cluster (page17).

3. How could outcome assessors be blinded to the condition assignment?

Response: Thank you for the comment. Is it corrected and explained at the text: „Outcome assessors and data analysts, with the exception of the principal investigator and the tester, were blinded to the condition assignment throughout the study, blinding was maintained until all measurements were completed and the dataset was fully prepared for statistical analysis.“

4.Add the number of men and women to Table 2.

Response: The number of men and women si added.

5. Is it gender differences or sex differences - in some places you say gender, in some sex, and you use males and females - please be consistent.

Response: Thank you. Everything is consistend now.

6. Given your focus on sex - your models should include an interaction with sex - it is not just that there is e.g. that there is a greater RCp for women than men - is the mask effect different for women than men.

Response: Thank you for your comment. The interaction effect between the respirator and gender was also incorporated into the model. Since this difference is insignificant for all breath segments, this interaction effect was not reflected in the resulting model due to the lack of overparameterization of the model.

7. Limitations should further acknowledge the very small sample size for sex specific results.

Response: Yes, it is true. That is added to work limits.
