# Supplementary material for: Respiratory pattern change in female and male runners by respiratory tract restriction using a respirator
Source: PLoS One. 2026 Jul 16;21(7):e0353784. doi: 10.1371/journal.pone.0353784 (PMC13374970; doi:10.1371/journal.pone.0353784)
Supplement: Attachment — Submitted filename: Response to review.docx [file pone.0353784.s006.docx]

Respiratory pattern change in female and male runners by respiratory tract restriction using a respirator

**Response to Reviewers**

Reviewer #1:

Point 1: I actually think that you are assessing sex differences - not gender. In the text you use gender, in the model you use sex. Probably should change all to sex.

Response 1: Thank you for the comment. Both text and figure have been corrected.

Reviewer #2:

Point 1: The paper is innovative because it investigates parameters that have been little explored in the literature considering the use of face mask during running. However, the justification could be better substantiated. Although it clearly states the influence of mask use on respiratory parameters, the introduction does not clearly explain the importance of analyzing the thoracoabdominal breathing pattern. I suggest including a paragraph highlighting this importance more emphatically.

Response 1: A paragraph has been added to the introduction to approximate this issue.

Point 2: The authors use “breath sector” instead of compartments. Considering that thoracoabdominal compartments or chestwall compartments are more common used in the classical literature, why to use breath sector?

Response 2: The word "sector" has been replaced by "compartment".

Point 3: Line 304-305: “When compared to untrained individuals, the runners in our study displayed significant shifts in thoracoabdominal activity during exercise, particularly through enhanced Ab and RCa activation.” – Clarify that the comparison is made based on data from the literature (and provide the reference), since the present study did no evaluate untrained individuals.

Response 3: Thank you for your comments. Yes, it is true, I added a citation.

Point 4: Besides the limitation considering the small sample size regarding gender comparisons, I suggest pointing out also the small sample size in the last phases of the tests. Considering that differences were found specifically in these phases, it could be important to comment on that.

Response 4: Limitation has been added to the text.

Point 5: Line 337-338: “The results suggest that highly trained individuals may be better adapted to acute respiratory stress compared to the general population.” – I suggest not concluding about the general population, since this was not expressed by the results (general population was not measured in the study). The conclusion must be supported by the results of the present study only.

Response 5: Yes, it is true. We have removed that sentence from the conclusion.
