# Supplementary material for: Respiratory pattern change in female and male runners by respiratory tract restriction using a respirator
Source: PLoS One. 2026 Jul 16;21(7):e0353784. doi: 10.1371/journal.pone.0353784 (PMC13374970; doi:10.1371/journal.pone.0353784)
Supplement: Peer review history

# Peer review: Decision Letter 0

**DOI:** 10.1371/journal.pone.0353784.r001

**Authors:** Ozeas de Lima Lins-Filho

8 Jan 2026

PONE-D-25-61828

Respiratory pattern change in female and male runners by respiratory tract restriction using a respirator

PLOS One

Dear Dr. Bahenský,

Thank you for submitting your manuscript to PLOS ONE. After careful consideration, we feel that it has merit but does not fully meet PLOS ONE’s publication criteria as it currently stands. Therefore, we invite you to submit a revised version of the manuscript that addresses the points raised during the review process.

Please submit your revised manuscript by Feb 22 2026 11:59PM. If you will need more time than this to complete your revisions, please reply to this message or contact the journal office at plosone@plos.org. When you're ready to submit your revision, log on to https://www.editorialmanager.com/pone/ and select the 'Submissions Needing Revision' folder to locate your manuscript file.

Please include the following items when submitting your revised manuscript:

- A letter that responds to each point raised by the academic editor and reviewer(s). You should upload this letter as a separate file labeled 'Response to Reviewers'.
- A marked-up copy of your manuscript that highlights changes made to the original version. You should upload this as a separate file labeled 'Revised Manuscript with Track Changes'.
- An unmarked version of your revised paper without tracked changes. You should upload this as a separate file labeled 'Manuscript'.

If you would like to make changes to your financial disclosure, please include your updated statement in your cover letter. Guidelines for resubmitting your figure files are available below the reviewer comments at the end of this letter.

If applicable, we recommend that you deposit your laboratory protocols in protocols.io to enhance the reproducibility of your results. Protocols.io assigns your protocol its own identifier (DOI) so that it can be cited independently in the future. For instructions see: https://journals.plos.org/plosone/s/submission-guidelines#loc-laboratory-protocols. Additionally, PLOS ONE offers an option for publishing peer-reviewed Lab Protocol articles, which describe protocols hosted on protocols.io. Read more information on sharing protocols at https://plos.org/protocols?utm_medium=editorial-email&utm_source=authorletters&utm_campaign=protocols.

We look forward to receiving your revised manuscript.

Kind regards,

Ozeas de Lima Lins-Filho, Ph.D.

Academic Editor

PLOS One

Journal Requirements:

When submitting your revision, we need you to address these additional requirements.

1. Please ensure that your manuscript meets PLOS ONE's style requirements, including those for file naming. The PLOS ONE style templates can be found at

https://journals.plos.org/plosone/s/file?id=wjVg/PLOSOne_formatting_sample_main_body.pdf and

https://journals.plos.org/plosone/s/file?id=ba62/PLOSOne_formatting_sample_title_authors_affiliations.pdf

2.  We note that you have selected “Clinical Trial” as your article type. PLOS ONE requires that all clinical trials are registered in an appropriate registry (the WHO list of approved registries is at https://www.who.int/clinical-trials-registry-platform/network/primary-registries" https://www.who.int/clinical-trials-registry-platform/network/primary-registries and more information on trial registration is at http://www.icmje.org/about-icmje/faqs/clinical-trials-registration/). Please state the name of the registry and the registration number (e.g. ISRCTN or ClinicalTrials.gov) in the submission data and on the title page of your manuscript. a) Please provide the complete date range for participant recruitment and follow-up in the methods section of your manuscript. b) If you have not yet registered your trial in an appropriate registry, we now require you to do so and will need confirmation of the trial registry number before we can pass your paper to the next stage of review. Please include in the Methods section of your paper your reasons for not registering this study before enrolment of participants started. Please confirm that all related trials are registered by stating: “The authors confirm that all ongoing and related trials for this drug/intervention are registered”. Please see http://journals.plos.org/plosone/s/submission-guidelines#loc-clinical-trials for our policies on clinical trials.

3. We note that the original protocol that you have uploaded as a Supporting Information file contains an institutional logo. As this logo is likely copyrighted, we ask that you please remove it from this file and upload an updated version upon resubmission.

4. If the reviewer comments include a recommendation to cite specific previously published works, please review and evaluate these publications to determine whether they are relevant and should be cited. There is no requirement to cite these works unless the editor has indicated otherwise.

[Note: HTML markup is below. Please do not edit.]

Reviewers' comments:

Reviewer's Responses to Questions

**Comments to the Author**

1. Is the manuscript technically sound, and do the data support the conclusions?

The manuscript must describe a technically sound piece of scientific research with data that supports the conclusions. Experiments must have been conducted rigorously, with appropriate controls, replication, and sample sizes. The conclusions must be drawn appropriately based on the data presented.

Reviewer #1: Yes

**********

2. Has the statistical analysis been performed appropriately and rigorously?

Reviewer #1: Yes

**********

3. Have the authors made all data underlying the findings in their manuscript fully available?

The PLOS Data policy requires authors to make all data underlying the findings described in their manuscript fully available without restriction, with rare exception (please refer to the Data Availability Statement in the manuscript PDF file). The data should be provided as part of the manuscript or its supporting information, or deposited to a public repository. For example, in addition to summary statistics, the data points behind means, medians and variance measures should be available. If there are restrictions on publicly sharing data—e.g. participant privacy or use of data from a third party—those must be specified.

Reviewer #1: No

**********

4. Is the manuscript presented in an intelligible fashion and written in standard English?

PLOS ONE does not copyedit accepted manuscripts, so the language in submitted articles must be clear, correct, and unambiguous. Any typographical or grammatical errors should be corrected at revision, so please note any specific errors here.

Reviewer #1: No

**********

5. Review Comments to the Author

Please use the space provided to explain your answers to the questions above. You may also include additional comments for the author, including concerns about dual publication, research ethics, or publication ethics. (Please upload your review as an attachment if it exceeds 20,000 characters)

Reviewer #1: THis paper looks at the effect of wearing an N95 mask during exercise on various respiratory parameters. Overall, grammar and phrasing need improvement.

1. In what setting were the runners recruited?

2. Please move table 1 and its description to the results section. In the methods section, please describe how this information was collected.

3. Your flow diagram makes no sense. For the first split, show how many were randomized to "mask" then "no mask" and how many to "no mask" then "mask".

4. Please use standard formulation to show the formula of the model, and not R code.

5. The language in lines 145-149 needs improvement.

6. "Finally, person is a random intercept model that allows us to model the correlation between data

148 gathered from the same person, i.e., the measurements of the same person with and without N95 respirator and in different

149 phases." Instead, "the model included a random intercept to model the correlation between observations from the same person."

7.Please remove the post-hoc sample size calculation. It provides no information beyond statistical significance.

8. Instead, please describe how the initial sample size was arrived at.

9. You do not describe the potential for sequence effects, period effects or carryover effects. Please add methods and results to address each of these.

11. Table 4 and 5 are hard to understand. Instead, show the RCP estimates in masked and non-masked situations with 95% CIs (no t-test value, no p value). Also present the p value for the interaction (the p value from Table 5). Probably better to show as a figure.

12. Please consider the possibility of modeling phase as a linear term?

13.Please discuss the possibility of informative cluster sizes on your analysis and the potential effects on the results observed.

14. In the discussion you say "sex relateddifferences were observed in ventiliatory responses...." To assess this properly, you need to include interactions with sex.

**********

6. PLOS authors have the option to publish the peer review history of their article (what does this mean?). If published, this will include your full peer review and any attached files.

If you choose “no”, your identity will remain anonymous but your review may still be made public.

**Do you want your identity to be public for this peer review?**  For information about this choice, including consent withdrawal, please see our Privacy Policy.

Reviewer #1: No

**********

[NOTE: If reviewer comments were submitted as an attachment file, they will be attached to this email and accessible via the submission site. Please log into your account, locate the manuscript record, and check for the action link "View Attachments". If this link does not appear, there are no attachment files.]

To ensure your figures meet our technical requirements, please review our figure guidelines: https://journals.plos.org/plosone/s/figures

You may also use PLOS’s free figure tool, NAAS, to help you prepare publication quality figures: https://journals.plos.org/plosone/s/figures#loc-tools-for-figure-preparation.

NAAS will assess whether your figures meet our technical requirements by comparing each figure against our figure specifications.

# Author comment: Author response to Decision Letter 1

**DOI:** 10.1371/journal.pone.0353784.r002

7 Feb 2026

1. Please ensure that your manuscript meets PLOS ONE's style requirements, including those for file naming. The PLOS ONE style templates can be found at

https://journals.plos.org/plosone/s/file?id=wjVg/PLOSOne_formatting_sample_main_body.pdf and

https://journals.plos.org/plosone/s/file?id=ba62/PLOSOne_formatting_sample_title_authors_affiliations.pdf

Response: I checked

2. We note that you have selected “Clinical Trial” as your article type. PLOS ONE requires that all clinical trials are registered in an appropriate registry (the WHO list of approved registries is at "https://www.who.int/clinical-trials-registry-platform/network/primary-registries" https://www.who.int/clinical-trials-registry-platform/network/primary-registries and more information on trial registration is at http://www.icmje.org/about-icmje/faqs/clinical-trials-registration/). Please state the name of the registry and the registration number (e.g. ISRCTN or ClinicalTrials.gov) in the submission data and on the title page of your manuscript. a) Please provide the complete date range for participant recruitment and follow-up in the methods section of your manuscript. b) If you have not yet registered your trial in an appropriate registry, we now require you to do so and will need confirmation of the trial registry number before we can pass your paper to the next stage of review. Please include in the Methods section of your paper your reasons for not registering this study before enrolment of participants started. Please confirm that all related trials are registered by stating: “The authors confirm that all ongoing and related trials for this drug/intervention are registered”. Please see http://journals.plos.org/plosone/s/submission-guidelines#loc-clinical-trials for our policies on clinical trials.

Response: The study is registered, the necessary information is given on the first page of the manuscript as well as in the Methods section, chapter 2.2.

3. We note that the original protocol that you have uploaded as a Supporting Information file contains an institutional logo. As this logo is likely copyrighted, we ask that you please remove it from this file and upload an updated version upon resubmission.

Response: We uploaded forms without the university logo into the system.

4. If the reviewer comments include a recommendation to cite specific previously published works, please review and evaluate these publications to determine whether they are relevant and should be cited. There is no requirement to cite these works unless the editor has indicated otherwise.

Response: Thank you.

Reviewers' comments:

1. Is the manuscript technically sound, and do the data support the conclusions?

The manuscript must describe a technically sound piece of scientific research with data that supports the conclusions. Experiments must have been conducted rigorously, with appropriate controls, replication, and sample sizes. The conclusions must be drawn appropriately based on the data presented.

Reviewer #1: Yes

2. Has the statistical analysis been performed appropriately and rigorously?

Reviewer #1: Yes

3. Have the authors made all data underlying the findings in their manuscript fully available?

The PLOS Data policy requires authors to make all data underlying the findings described in their manuscript fully available without restriction, with rare exception (please refer to the Data Availability Statement in the manuscript PDF file). The data should be provided as part of the manuscript or its supporting information, or deposited to a public repository. For example, in addition to summary statistics, the data points behind means, medians and variance measures should be available. If there are restrictions on publicly sharing data—e.g. participant privacy or use of data from a third party—those must be specified.

Reviewer #1: No

Response: The data is available on the website: http://www.petrbahensky.wz.cz/pdf/Conference010.pdf

This information is provided in the article in Chapter 2.6

4. Is the manuscript presented in an intelligible fashion and written in standard English?

PLOS ONE does not copyedit accepted manuscripts, so the language in submitted articles must be clear, correct, and unambiguous. Any typographical or grammatical errors should be corrected at revision, so please note any specific errors here.

Reviewer #1: No

Response: One of the authors is a native speaker, he checked and edited the text.

5. Review Comments to the Author

Please use the space provided to explain your answers to the questions above. You may also include additional comments for the author, including concerns about dual publication, research ethics, or publication ethics. (Please upload your review as an attachment if it exceeds 20,000 characters)

Reviewer #1: THis paper looks at the effect of wearing an N95 mask during exercise on various respiratory parameters. Overall, grammar and phrasing need improvement.

Response: Thank you for your comment, we have edited the text, one of the authors is a native speaker.

1. In what setting were the runners recruited?

Response: Thank you for your comment. The runners were recruited through local athletics clubs in the South Bohemia region. Coaches were contacted and informed about the study, and eligible athletes were subsequently invited to participate on a voluntary basis.

2. Please move table 1 and its description to the results section. In the methods section, please describe how this information was collected.

Response: Thank you for your comment, I moved table 1 to the results section (it is currently table 2) and in the methodology section I described the data collection.

3. Your flow diagram makes no sense. For the first split, show how many were randomized to "mask" then "no mask" and how many to "no mask" then "mask".

Response: Thank you for your comment. Yes, really the diagram was not correct. We created a new one, which we inserted into the text.

4. Please use standard formulation to show the formula of the model, and not R code.

Response: Thank you for your comment. We have edited the formula and commentary. The paragraph was rewritten in the following way:

To analyze the data was performed the linear mixed-effect model (LM) [42] with the formula:

RCp_{ij} = \beta_0+ \beta_1 (Sex_j )+ \beta_2 (Phase_{ij} )+ \beta_3 (Phase_{ij} ×Respirator_{ij} )+ u_{0j} + ϵ_{ij}

Here, Sex and Phase represent standard fixed effects. The term Phase × Respirator denotes the fixed interaction effect, which allows us to estimate the difference associated with using the N95 respirator separately for each phase. The term u_{0j} represents the random intercept for every person j; this accounts for the correlation between repeated measurements gathered from the same individual (i.e., measurements taken with and without the N95 respirator across different phases). Finally, the β coefficients denote the model parameters corresponding to each effect, and ϵ_{ij} represents the residual error.

5. The language in lines 145-149 needs improvement.

Response: Thank you for your comment. See above point. The text was rewritten by one of the authors - a native speaker.

6. "Finally, person is a random intercept model that allows us to model the correlation between data

148 gathered from the same person, i.e., the measurements of the same person with and without N95 respirator and in different

149 phases." Instead, "the model included a random intercept to model the correlation between observations from the same person."

Response: Thank you for your comment. We rewrote this sentence a bit differently due to the rewriting of the formula.

7.Please remove the post-hoc sample size calculation. It provides no information beyond statistical significance.

Response: Thank you for your comment. Point 2.5 has been deleted.

8. Instead, please describe how the initial sample size was arrived at.

Response: Thank you for your comment. In order to obtain credible results, we worked with a very homogeneous group of participants (i.e., top athletes, of a particular age), therefore we do not expect any subgroups of athletes in the data base that could distort the results, and therefore our data sample contains only 20 participants, all who met the criteria. We stated this in paragraph 2.1.

9. You do not describe the potential for sequence effects, period effects or carryover effects. Please add methods and results to address each of these.

Response: Thank you for your comment. The order of the tests was randomized, with half of the participants taking the first test with a respirator and half of the participants taking the test without a respirator. We have added to the text in chapter 2.3.1.

11. Table 4 and 5 are hard to understand. Instead, show the RCP estimates in masked and non-masked situations with 95% CIs (no t-test value, no p value). Also present the p value for the interaction (the p value from Table 5). Probably better to show as a figure.

Response: Thank you for your comment. We summarize the results according your advice in the following figures. We have replaced the tables with these figures:

12. Please consider the possibility of modeling phase as a linear term?

Response: Thank you for your comment. Our goal was to determine how the breath pattern changes, particularly when the behavior changes, and what changes occurred in the different phases. We believe that converting the phase to a linear trend would simplify the problem and introduce the assumption of linearity, which seems defensible for the first phases but not for the later phases, when the patterning appears to have already stabilized. We would find out how the pattern changes assuming linearity, but we wouldn't find out when the pattern is already different.

13.Please discuss the possibility of informative cluster sizes on your analysis and the potential effects on the results observed.

Response: See the point 8 - Thank you for your comment. In order to obtain credible results, we worked with a very homogeneous group of participants (i.e., top athletes, of a particular age), therefore we do not expect any subgroups of athletes in the data base that could distort the results, and therefore our data sample contains only 20 participants.

14. In the discussion you say "sex relateddifferences were observed in ventiliatory responses...." To assess this properly, you need to include interactions with sex.

Response: Thank you for your comment. Originally, we did not assume that the respirator would have a significantly different effect on men and women. Nevertheless, we included the intersex differences in the analysis: Figure 4, Figure 7 (originally Table 4). But we modified the statement in the discussion to: „Notably observed, sex-specific trends were observed in ventilatory responses…“

Attachment Submitted filename: Response to Reviewers.docx

# Peer review: Decision Letter 1

**DOI:** 10.1371/journal.pone.0353784.r003

**Authors:** Ozeas de Lima Lins-Filho

22 Feb 2026

PONE-D-25-61828R1

Respiratory pattern change in female and male runners by respiratory tract restriction using a respirator

PLOS One

Dear Dr. Bahenský,

Thank you for submitting your manuscript to PLOS ONE. After careful consideration, we feel that it has merit but does not fully meet PLOS ONE’s publication criteria as it currently stands. Therefore, we invite you to submit a revised version of the manuscript that addresses the points raised during the review process.

Please submit your revised manuscript by Apr 08 2026 11:59PM. If you will need more time than this to complete your revisions, please reply to this message or contact the journal office at plosone@plos.org. When you're ready to submit your revision, log on to https://www.editorialmanager.com/pone/ and select the 'Submissions Needing Revision' folder to locate your manuscript file.

Please include the following items when submitting your revised manuscript:

- A letter that responds to each point raised by the academic editor and reviewer(s). You should upload this letter as a separate file labeled 'Response to Reviewers'.
- A marked-up copy of your manuscript that highlights changes made to the original version. You should upload this as a separate file labeled 'Revised Manuscript with Track Changes'.
- An unmarked version of your revised paper without tracked changes. You should upload this as a separate file labeled 'Manuscript'.

If you would like to make changes to your financial disclosure, please include your updated statement in your cover letter. Guidelines for resubmitting your figure files are available below the reviewer comments at the end of this letter.

If applicable, we recommend that you deposit your laboratory protocols in protocols.io to enhance the reproducibility of your results. Protocols.io assigns your protocol its own identifier (DOI) so that it can be cited independently in the future. For instructions see: https://journals.plos.org/plosone/s/submission-guidelines#loc-laboratory-protocols. Additionally, PLOS ONE offers an option for publishing peer-reviewed Lab Protocol articles, which describe protocols hosted on protocols.io. Read more information on sharing protocols at https://plos.org/protocols?utm_medium=editorial-email&utm_source=authorletters&utm_campaign=protocols.

We look forward to receiving your revised manuscript.

Kind regards,

Ozeas de Lima Lins-Filho, Ph.D.

Academic Editor

PLOS One

**Journal Requirements:**

If the reviewer comments include a recommendation to cite specific previously published works, please review and evaluate these publications to determine whether they are relevant and should be cited. There is no requirement to cite these works unless the editor has indicated otherwise.

[Note: HTML markup is below. Please do not edit.]

Reviewers' comments:

Reviewer's Responses to Questions

**Comments to the Author**

1. If the authors have adequately addressed your comments raised in a previous round of review and you feel that this manuscript is now acceptable for publication, you may indicate that here to bypass the “Comments to the Author” section, enter your conflict of interest statement in the “Confidential to Editor” section, and submit your "Accept" recommendation.

Reviewer #1: (No Response)

**********

2. Is the manuscript technically sound, and do the data support the conclusions?

The manuscript must describe a technically sound piece of scientific research with data that supports the conclusions. Experiments must have been conducted rigorously, with appropriate controls, replication, and sample sizes. The conclusions must be drawn appropriately based on the data presented.

Reviewer #1: Partly

**********

3. Has the statistical analysis been performed appropriately and rigorously?

Reviewer #1: No

**********

4. Have the authors made all data underlying the findings in their manuscript fully available?

The PLOS Data policy requires authors to make all data underlying the findings described in their manuscript fully available without restriction, with rare exception (please refer to the Data Availability Statement in the manuscript PDF file). The data should be provided as part of the manuscript or its supporting information, or deposited to a public repository. For example, in addition to summary statistics, the data points behind means, medians and variance measures should be available. If there are restrictions on publicly sharing data—e.g. participant privacy or use of data from a third party—those must be specified.

Reviewer #1: Yes

**********

5. Is the manuscript presented in an intelligible fashion and written in standard English?

PLOS ONE does not copyedit accepted manuscripts, so the language in submitted articles must be clear, correct, and unambiguous. Any typographical or grammatical errors should be corrected at revision, so please note any specific errors here.

Reviewer #1: Yes

**********

6. Review Comments to the Author

Please use the space provided to explain your answers to the questions above. You may also include additional comments for the author, including concerns about dual publication, research ethics, or publication ethics. (Please upload your review as an attachment if it exceeds 20,000 characters)

Reviewer #1: 1. Your response does not address this comment: You do not describe the potential for sequence effects, period effects or carryover

effects. Please add methods and results to address each of these.

Response: Thank you for your comment. The order of the tests was randomized, with

half of the participants taking the first test with a respirator and half of the participants

taking the test without a respirator. We have added to the text in chapter 2.3.1.

2. Your response does not adddress this comment: Please discuss the possibility of informative cluster sizes on your analysis and the

potential effects on the results observed.

Response: See the point 8 - Thank you for your comment. In order to obtain credible results, we worked with a very homogeneous group of participants (i.e., top athletes, of a particular age), therefore we do not expect any subgroups of athletes in the data base that could distort the results, and therefore our data sample contains only 20

participants

3. How could outcome assessors be blinded to the condition assignment?

4.Add the number of men and women to Table 2.

5. Is it gender differences or sex differences - in some places you say gender, in some sex, and you use males and females - please be consistent.

6. Given your focus on sex - your models should include an interaction with sex - it is not just that there is e.g. that there is a greater RCp for women than men - is the mask effect different for women than men.

7. Limitations should further acknowledge the very small sample size for sex specific results.

**********

7. PLOS authors have the option to publish the peer review history of their article (what does this mean?). If published, this will include your full peer review and any attached files.

If you choose “no”, your identity will remain anonymous but your review may still be made public.

**Do you want your identity to be public for this peer review?**  For information about this choice, including consent withdrawal, please see our Privacy Policy.

Reviewer #1: No

**********

[NOTE: If reviewer comments were submitted as an attachment file, they will be attached to this email and accessible via the submission site. Please log into your account, locate the manuscript record, and check for the action link "View Attachments". If this link does not appear, there are no attachment files.]

To ensure your figures meet our technical requirements, please review our figure guidelines: https://journals.plos.org/plosone/s/figures

You may also use PLOS’s free figure tool, NAAS, to help you prepare publication quality figures: https://journals.plos.org/plosone/s/figures#loc-tools-for-figure-preparation.

NAAS will assess whether your figures meet our technical requirements by comparing each figure against our figure specifications.

# Author comment: Author response to Decision Letter 2

**DOI:** 10.1371/journal.pone.0353784.r004

5 Mar 2026

1. Your response does not address this comment: You do not describe the potential for sequence effects, period effects or carryover effects. Please add methods and results to address each of these.

Response: We apologize to the reviewer that we were not specific considering some of his/her comment.

We incorporated the sequance effect, period effect and carryover effect into the modeling. The procedure and results are in the article on pages 16-17.

2. Your response does not adddress this comment: Please discuss the possibility of informative cluster sizes on your analysis and the potential effects on the results observed.

Response: We apologize to the reviewer that we were not specific considering some of his/her comment.

The size of clusters is considered "informational" if the number of observations in a given individual is directly related to the resulting value of the measured quantity. In the presented study, the size of the cluster is determined by how many phases of the Bruce Protocol the runner completed before reaching subjective exhaustion. According to Table 3 in the manuscript, the size of the clusters ranges from 4 to 6 stages (12 to 18 minutes). Exercisers who completed the test earlier are likely to have less lung capacity and therefore may experience a different compensation of breathing pattern than exercisers who completed the test later. However, due to the nature of the Bruce protocol, which ran until free exhaustion, there is a variable number of observations per participant in our data (clusters of 4 to 6 phases). This situation requires consideration of the phenomenon of information cluster size (ICS), where the duration of the test correlates with the physiological fitness of the subject. On a theoretical level, ICS could lead to over-influencing population averages by runners with the highest endurance. In our study, however, this risk is minimized by the high homogeneity of the training of the cohort and the fact that the primary goal – the evaluation of the effect of the respirator – was implemented as an intra-subject comparison within each cluster (page17).

3. How could outcome assessors be blinded to the condition assignment?

Response: Thank you for the comment. Is it corrected and explained at the text: „Outcome assessors and data analysts, with the exception of the principal investigator and the tester, were blinded to the condition assignment throughout the study, blinding was maintained until all measurements were completed and the dataset was fully prepared for statistical analysis.“

4.Add the number of men and women to Table 2.

Response: The number of men and women si added.

5. Is it gender differences or sex differences - in some places you say gender, in some sex, and you use males and females - please be consistent.

Response: Thank you. Everything is consistend now.

6. Given your focus on sex - your models should include an interaction with sex - it is not just that there is e.g. that there is a greater RCp for women than men - is the mask effect different for women than men.

Response: Thank you for your comment. The interaction effect between the respirator and gender was also incorporated into the model. Since this difference is insignificant for all breath segments, this interaction effect was not reflected in the resulting model due to the lack of overparameterization of the model.

7. Limitations should further acknowledge the very small sample size for sex specific results.

Response: Yes, it is true. That is added to work limits.

Attachment Submitted filename: Response to review last.docx

# Peer review: Decision Letter 2

**DOI:** 10.1371/journal.pone.0353784.r005

**Authors:** Ozeas de Lima Lins-Filho

22 Apr 2026

PONE-D-25-61828R2

Respiratory pattern change in female and male runners by respiratory tract restriction using a respirator

PLOS One

Dear Dr. Bahenský,

Thank you for submitting your manuscript to PLOS ONE. After careful consideration, we feel that it has merit but does not fully meet PLOS ONE’s publication criteria as it currently stands. Therefore, we invite you to submit a revised version of the manuscript that addresses the points raised during the review process.

==============================

Reviewer #1: I actually think that you are assessing sex differences - not gender. In the text you use gender, in the model you use sex. Probably should change all to sex.

Reviewer #2: The paper is innovative because it investigates parameters that have been little explored in the literature considering the use of face mask during running. However, the justification could be better substantiated. Although it clearly states the influence of mask use on respiratory parameters, the introduction does not clearly explain the importance of analyzing the thoracoabdominal breathing pattern. I suggest including a paragraph highlighting this importance more emphatically.

The authors use “breath sector” instead of compartments. Considering that thoracoabdominal compartments or chestwall compartments are more common used in the classical literature, why to use breath sector?

Line 304-305: “When compared to untrained individuals, the runners in our study displayed significant shifts in thoracoabdominal activity during exercise, particularly through enhanced Ab and RCa activation.” – Clarify that the comparison is made based on data from the literature (and provide the reference), since the present study did no evaluate untrained individuals.

Besides the limitation considering the small sample size regarding gender comparisons, I suggest pointing out also the small sample size in the last phases of the tests. Considering that differences were found specifically in these phases, it could be important to comment on that.

Line 337-338: “The results suggest that highly trained individuals may be better adapted to acute respiratory stress compared to the general population.” – I suggest not concluding about the general population, since this was not expressed by the results (general population was not measured in the study). The conclusion must be supported by the results of the present study only.

==============================

Please submit your revised manuscript by Jun 06 2026 11:59PM. If you will need more time than this to complete your revisions, please reply to this message or contact the journal office at plosone@plos.org. When you're ready to submit your revision, log on to https://www.editorialmanager.com/pone/ and select the 'Submissions Needing Revision' folder to locate your manuscript file.

Please include the following items when submitting your revised manuscript:

- A letter that responds to each point raised by the academic editor and reviewer(s). You should upload this letter as a separate file labeled 'Response to Reviewers'.
- A marked-up copy of your manuscript that highlights changes made to the original version. You should upload this as a separate file labeled 'Revised Manuscript with Track Changes'.
- An unmarked version of your revised paper without tracked changes. You should upload this as a separate file labeled 'Manuscript'.

If you would like to make changes to your financial disclosure, please include your updated statement in your cover letter. Guidelines for resubmitting your figure files are available below the reviewer comments at the end of this letter.

If applicable, we recommend that you deposit your laboratory protocols in protocols.io to enhance the reproducibility of your results. Protocols.io assigns your protocol its own identifier (DOI) so that it can be cited independently in the future. For instructions see: https://journals.plos.org/plosone/s/submission-guidelines#loc-laboratory-protocols. Additionally, PLOS ONE offers an option for publishing peer-reviewed Lab Protocol articles, which describe protocols hosted on protocols.io. Read more information on sharing protocols at https://plos.org/protocols?utm_medium=editorial-email&utm_source=authorletters&utm_campaign=protocols.

As the corresponding author, your ORCID iD is verified in the submission system and will appear in the published article. PLOS supports the use of ORCID, and we encourage all coauthors to register for an ORCID iD and use it as well. Please encourage your coauthors to verify their ORCID iD within the submission system before final acceptance, as unverified ORCID iDs will not appear in the published article. *Only*  the individual author can complete the verification step; PLOS staff *cannot*  verify ORCID iDs on behalf of authors.

We look forward to receiving your revised manuscript.

Kind regards,

Ozeas de Lima Lins-Filho, Ph.D.

Academic Editor

PLOS One

Journal Requirements:

1. If the reviewer comments include a recommendation to cite specific previously published works, please review and evaluate these publications to determine whether they are relevant and should be cited. There is no requirement to cite these works unless the editor has indicated otherwise.

2. Please review your reference list to ensure that it is complete and correct. If you have cited papers that have been retracted, please include the rationale for doing so in the manuscript text, or remove these references and replace them with relevant current references. Any changes to the reference list should be mentioned in the rebuttal letter that accompanies your revised manuscript. If you need to cite a retracted article, indicate the article’s retracted status in the References list and also include a citation and full reference for the retraction notice.

[Note: HTML markup is below. Please do not edit.]

Reviewers' comments:

Reviewer's Responses to Questions

**Comments to the Author**

1. If the authors have adequately addressed your comments raised in a previous round of review and you feel that this manuscript is now acceptable for publication, you may indicate that here to bypass the “Comments to the Author” section, enter your conflict of interest statement in the “Confidential to Editor” section, and submit your "Accept" recommendation.

Reviewer #1: All comments have been addressed

Reviewer #2: (No Response)

**********

2. Is the manuscript technically sound, and do the data support the conclusions?

The manuscript must describe a technically sound piece of scientific research with data that supports the conclusions. Experiments must have been conducted rigorously, with appropriate controls, replication, and sample sizes. The conclusions must be drawn appropriately based on the data presented.

Reviewer #1: Yes

Reviewer #2: Partly

**********

3. Has the statistical analysis been performed appropriately and rigorously?

Reviewer #1: Yes

Reviewer #2: I Don't Know

**********

4. Have the authors made all data underlying the findings in their manuscript fully available?

The PLOS Data policy requires authors to make all data underlying the findings described in their manuscript fully available without restriction, with rare exception (please refer to the Data Availability Statement in the manuscript PDF file). The data should be provided as part of the manuscript or its supporting information, or deposited to a public repository. For example, in addition to summary statistics, the data points behind means, medians and variance measures should be available. If there are restrictions on publicly sharing data—e.g. participant privacy or use of data from a third party—those must be specified.

Reviewer #1: Yes

Reviewer #2: Yes

**********

5. Is the manuscript presented in an intelligible fashion and written in standard English?

PLOS ONE does not copyedit accepted manuscripts, so the language in submitted articles must be clear, correct, and unambiguous. Any typographical or grammatical errors should be corrected at revision, so please note any specific errors here.

Reviewer #1: Yes

Reviewer #2: Yes

**********

6. Review Comments to the Author

-->Reviewer #1: I actually think that you are assessing sex differences - not gender. In the text you use gender, in the model you use sex. Probably should change all to sex.

Reviewer #2: The paper is innovative because it investigates parameters that have been little explored in the literature considering the use of face mask during running. However, the justification could be better substantiated. Although it clearly states the influence of mask use on respiratory parameters, the introduction does not clearly explain the importance of analyzing the thoracoabdominal breathing pattern. I suggest including a paragraph highlighting this importance more emphatically.

The authors use “breath sector” instead of compartments. Considering that thoracoabdominal compartments or chestwall compartments are more common used in the classical literature, why to use breath sector?

Line 304-305: “When compared to untrained individuals, the runners in our study displayed significant shifts in thoracoabdominal activity during exercise, particularly through enhanced Ab and RCa activation.” – Clarify that the comparison is made based on data from the literature (and provide the reference), since the present study did no evaluate untrained individuals.

Besides the limitation considering the small sample size regarding gender comparisons, I suggest pointing out also the small sample size in the last phases of the tests. Considering that differences were found specifically in these phases, it could be important to comment on that.

Line 337-338: “The results suggest that highly trained individuals may be better adapted to acute respiratory stress compared to the general population.” – I suggest not concluding about the general population, since this was not expressed by the results (general population was not measured in the study). The conclusion must be supported by the results of the present study only.

**********

7. PLOS authors have the option to publish the peer review history of their article (what does this mean?). If published, this will include your full peer review and any attached files.

If you choose “no”, your identity will remain anonymous but your review may still be made public.

**Do you want your identity to be public for this peer review?**  For information about this choice, including consent withdrawal, please see our Privacy Policy.

Reviewer #1: No

Reviewer #2: No

**********

[NOTE: If reviewer comments were submitted as an attachment file, they will be attached to this email and accessible via the submission site. Please log into your account, locate the manuscript record, and check for the action link "View Attachments". If this link does not appear, there are no attachment files.]

To ensure your figures meet our technical requirements, please review our figure guidelines: https://journals.plos.org/plosone/s/figures

You may also use PLOS’s free figure tool, NAAS, to help you prepare publication quality figures: https://journals.plos.org/plosone/s/figures#loc-tools-for-figure-preparation.

NAAS will assess whether your figures meet our technical requirements by comparing each figure against our figure specifications.

# Author comment: Author response to Decision Letter 3

**DOI:** 10.1371/journal.pone.0353784.r006

28 Apr 2026

Respiratory pattern change in female and male runners by respiratory tract restriction using a respirator

Response to Reviewers

Reviewer #1:

Point 1: I actually think that you are assessing sex differences - not gender. In the text you use gender, in the model you use sex. Probably should change all to sex.

Response 1: Thank you for the comment. Both text and figure have been corrected.

Reviewer #2:

Point 1: The paper is innovative because it investigates parameters that have been little explored in the literature considering the use of face mask during running. However, the justification could be better substantiated. Although it clearly states the influence of mask use on respiratory parameters, the introduction does not clearly explain the importance of analyzing the thoracoabdominal breathing pattern. I suggest including a paragraph highlighting this importance more emphatically.

Response 1: A paragraph has been added to the introduction to approximate this issue.

Point 2: The authors use “breath sector” instead of compartments. Considering that thoracoabdominal compartments or chestwall compartments are more common used in the classical literature, why to use breath sector?

Response 2: The word "sector" has been replaced by "compartment".

Point 3: Line 304-305: “When compared to untrained individuals, the runners in our study displayed significant shifts in thoracoabdominal activity during exercise, particularly through enhanced Ab and RCa activation.” – Clarify that the comparison is made based on data from the literature (and provide the reference), since the present study did no evaluate untrained individuals.

Response 3: Thank you for your comments. Yes, it is true, I added a citation.

Point 4: Besides the limitation considering the small sample size regarding gender comparisons, I suggest pointing out also the small sample size in the last phases of the tests. Considering that differences were found specifically in these phases, it could be important to comment on that.

Response 4: Limitation has been added to the text.

Point 5: Line 337-338: “The results suggest that highly trained individuals may be better adapted to acute respiratory stress compared to the general population.” – I suggest not concluding about the general population, since this was not expressed by the results (general population was not measured in the study). The conclusion must be supported by the results of the present study only.

Response 5: Yes, it is true. We have removed that sentence from the conclusion.

Attachment Submitted filename: Response to review.docx

# Peer review: Decision Letter 3

**DOI:** 10.1371/journal.pone.0353784.r007

**Authors:** Ozeas de Lima Lins-Filho

30 Jun 2026

Respiratory pattern change in female and male runners by respiratory tract restriction using a respirator

PONE-D-25-61828R3

Dear Dr. Bahenský,

We’re pleased to inform you that your manuscript has been judged scientifically suitable for publication and will be formally accepted for publication once it meets all outstanding technical requirements.

Within one week, you’ll receive an e-mail detailing the required amendments. When these have been addressed, you’ll receive a formal acceptance letter and your manuscript will be scheduled for publication.

An invoice will be generated when your article is formally accepted. Please note, if your institution has a publishing partnership with PLOS and your article meets the relevant criteria, all or part of your publication costs will be covered. Please make sure your user information is up-to-date by logging into Editorial Manager at Editorial Manager® and clicking the ‘Update My Information' link at the top of the page. For questions related to billing, please contact billing support.

If your institution or institutions have a press office, please notify them about your upcoming paper to help maximize its impact. If they’ll be preparing press materials, please inform our press team as soon as possible -- no later than 48 hours after receiving the formal acceptance. Your manuscript will remain under strict press embargo until 2 pm Eastern Time on the date of publication. For more information, please contact onepress@plos.org.

Kind regards,

Ozeas de Lima Lins-Filho, Ph.D.

Academic Editor

PLOS One

Additional Editor Comments (optional):

Reviewers' comments:

Reviewer's Responses to Questions

**Comments to the Author**

1. If the authors have adequately addressed your comments raised in a previous round of review and you feel that this manuscript is now acceptable for publication, you may indicate that here to bypass the “Comments to the Author” section, enter your conflict of interest statement in the “Confidential to Editor” section, and submit your "Accept" recommendation.

Reviewer #1: All comments have been addressed

Reviewer #2: All comments have been addressed

**********

2. Is the manuscript technically sound, and do the data support the conclusions?

The manuscript must describe a technically sound piece of scientific research with data that supports the conclusions. Experiments must have been conducted rigorously, with appropriate controls, replication, and sample sizes. The conclusions must be drawn appropriately based on the data presented.

Reviewer #1: Yes

Reviewer #2: Yes

**********

3. Has the statistical analysis been performed appropriately and rigorously?

Reviewer #1: Yes

Reviewer #2: Yes

**********

4. Have the authors made all data underlying the findings in their manuscript fully available?

The PLOS Data policy requires authors to make all data underlying the findings described in their manuscript fully available without restriction, with rare exception (please refer to the Data Availability Statement in the manuscript PDF file). The data should be provided as part of the manuscript or its supporting information, or deposited to a public repository. For example, in addition to summary statistics, the data points behind means, medians and variance measures should be available. If there are restrictions on publicly sharing data—e.g. participant privacy or use of data from a third party—those must be specified.

Reviewer #1: Yes

Reviewer #2: Yes

**********

5. Is the manuscript presented in an intelligible fashion and written in standard English?

PLOS ONE does not copyedit accepted manuscripts, so the language in submitted articles must be clear, correct, and unambiguous. Any typographical or grammatical errors should be corrected at revision, so please note any specific errors here.

Reviewer #1: Yes

Reviewer #2: Yes

**********

6. Review Comments to the Author

Please use the space provided to explain your answers to the questions above. You may also include additional comments for the author, including concerns about dual publication, research ethics, or publication ethics. (Please upload your review as an attachment if it exceeds 20,000 characters)

Reviewer #1: (No Response)

Reviewer #2: All previous comments have been answered satisfactorily. The authors have addressed every point thoroughly, and the manuscript has improved significantly. Consequently, there are no further comments or revisions required at this stage.

**********

7. PLOS authors have the option to publish the peer review history of their article (what does this mean?). If published, this will include your full peer review and any attached files.

If you choose “no”, your identity will remain anonymous but your review may still be made public.

**Do you want your identity to be public for this peer review?**  For information about this choice, including consent withdrawal, please see our Privacy Policy.

Reviewer #1: No

Reviewer #2: **Yes:;** Karine Jacon Sarro

**********

# Editor report: Acceptance letter

**DOI:** 10.1371/journal.pone.0353784.r008

**Authors:** Ozeas de Lima Lins-Filho

PONE-D-25-61828R3

PLOS One

Dear Dr. Bahenský,

I'm pleased to inform you that your manuscript has been deemed suitable for publication in PLOS One. Congratulations! Your manuscript is now being handed over to our production team.

At this stage, our production department will prepare your paper for publication. This includes ensuring the following:

* All references, tables, and figures are properly cited

* All relevant supporting information is included in the manuscript submission,

* There are no issues that prevent the paper from being properly typeset

You will receive further instructions from the production team, including instructions on how to review your proof when it is ready. Please keep in mind that we are working through a large volume of accepted articles, so please give us a few days to review your paper and let you know the next and final steps.

Lastly, if your institution or institutions have a press office, please let them know about your upcoming paper now to help maximize its impact. If they'll be preparing press materials, please inform our press team within the next 48 hours. Your manuscript will remain under strict press embargo until 2 pm Eastern Time on the date of publication. For more information, please contact onepress@plos.org.

You will receive an invoice from PLOS for your publication fee after your manuscript has reached the completed accept phase. If you receive an email requesting payment before acceptance or for any other service, this may be a phishing scheme. Learn how to identify phishing emails and protect your accounts at https://explore.plos.org/phishing.

If we can help with anything else, please email us at customercare@plos.org.

Thank you for submitting your work to PLOS ONE and supporting open access.

Kind regards,

PLOS ONE Editorial Office Staff

on behalf of

Dr. Ozeas de Lima Lins-Filho

Academic Editor

PLOS One
